# Coordinative‐Guest‐Triggered Dimensional Crossover in a Metal–Organic Framework With Rigid Metal–Oxo Rods

**DOI:** 10.1002/chem.71203

**Published:** 2026-06-01

**Authors:** Keisuke Ishikawa, Kazuya Otsubo

**Affiliations:** ^1^ Department of Chemistry Faculty of Science Tokyo University of Science Tokyo Japan; ^2^ Research Institute for Science and Technology Division of Joint Research of Geometry and Natural Science Tokyo University of Science Chiba Japan

**Keywords:** host‐guest systems, lead, metal‐organic frameworks, microporous materials, phase transitions

## Abstract

Structural phase transitions of metal–organic frameworks (MOFs) induced by guest‐molecule exchange within their pores have attracted considerable attention not only from the viewpoint of structural chemistry but also for the modulation of their functional properties. Herein, we report a reversible structural phase transition triggered by the exchange of coordinative guest molecules in a Pb‐based MOF composed of Pb^2+^ ions and 2,5‐dihydroxy‐1,4‐benzoquinone (H_2_DHBQ), formulated as [Pb(DHBQ)(DMF)(H_2_O)] (DMF = *N*,*N*‐dimethylformamide). This compound forms a 3D framework structure in which 1D Pb–O rods, constructed from Pb^2+^ ions and oxygen atoms of bridging DHBQ^2−^ ligands, serve as secondary building units (SBUs). The framework contains 1D pores accommodating coordinative DMF and H_2_O molecules. Exchange of these guest molecules induces a reversible structural phase transition accompanied by significant changes in both the Pb–O rod structure and the overall dimensionality of the framework. To the best of our knowledge, this represents the first example of a MOF composed of rigid SBUs that exhibits a reversible structural phase transition involving a change in framework dimensionality driven by guest‐molecule exchange. These findings provide new insight into dynamic structural transformations in MOFs and highlight a strategy for controlling framework topology and properties through guest coordination.

## Graphical abstract

We report a reversible structural phase transition in a metal–organic framework (MOF) triggered by the exchange of coordinative guest molecules. This MOF consists of rigid 1D Pb–O rods as building units, forming a 3D framework with 1D pores. Guest exchange induces pronounced changes in both the Pb–O rod structure and the dimensionality of the framework. This represents the first example of a MOF with rigid building units exhibiting a reversible dimensional transformation driven by guest exchange.

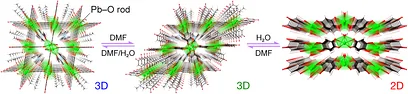

## 1 Introduction

Metal–organic frameworks (MOFs), which are made through the self‐assembly of diverse combinations of metal ions and organic ligands, have established a wide research field because of their wide range of potential applications [1, 2]. These include gas adsorption/separation [3, 4] and storage [5] utilizing their regular pores, catalytic reactions [6], proton/electron conduction [7, 8], sensor materials [9, 10], and biomedical applications [11]. The highly diverse network structures found in MOFs can be understood in terms of the combination of secondary building units (SBUs)—structural units formed from the coordination bonding sites of metal ions and ligands—and organic linkers possessing a variety of geometric shapes [12]. Among SBUs, those consisting of 1D infinite chain structures formed by the linkage of polyhedral units composed of metal ions and portions of organic ligands are generally referred to as 1D rods or 1D chains, and numerous MOFs incorporating such SBUs have been reported [13, 14, 15, 16]. Among 1D rod structures, the infinite chain structures formed by bridging metal ions with oxygen atoms derived from carboxyl or hydroxyl groups in organic ligands are specifically referred to as metal–oxo (M–O) rods [17, 18, 19, 20, 21, 22, 23, 24, 25, 26, 27, 28, 29, 30, 31, 32, 33, 34]. Well‐known examples of MOFs that possess M–O rods as SBUs include MOF‐74 [35], which consists of Zn–O rods formed from Zn^2+^ ions and 2,5‐dihydroxybenzene‐1,4‐dicarboxylic acid, and MIL‐53 [36], which is composed of Cr–O rods constructed from Cr^3+^ ions and benzene‐1,4‐dicarboxylic acid.

Besides, MOFs possess a major characteristic compared with conventional porous materials, such as zeolites, in that their structures are relatively flexible due to their composition from organic ligands [37]. Owing to this structural flexibility, several examples have been reported in which the crystal structure undergoes a transformation through the exchange of coordinating or noncoordinating guest molecules incorporated within the pores. Examples in which the dimensionality of a crystal structure changes through molecular exchange within MOFs have been reported, although most are irreversible; nevertheless, a few systems exhibiting reversible changes in dimensionality are known [38, 39, 40, 41, 42, 43, 44, 45, 46, 47, 48]. For example, reversible structural transformations have been reported in which the exchange of water (H_2_O) and *N*,*N*‐dimethylformamide (DMF) molecules converts the 1D structure [Cu(BDC)(H_2_O)_2_]·H_2_O into the 2D structure [Cu(BDC)(DMF)] or the 3D structure [Cu(BDC)] (BDC = 1,4‐benzenedicarboxylate), resulting in significant changes in gas adsorption and magnetic properties [49]. Another notable example is the reversible interconversion between the 1D structure [Ag_3_L_2_(CH_3_CN)_2_(OTf)_3_] and the 2D structure [Ag_3_L_2_(OTf)_3_] (L = diphenyl(2‐pyrimidyl)phosphine; OTf = trifluoromethanesulfonate) upon exposure to acetonitrile or dichloromethane vapor [50].

On the other hand, examples of guest‐molecule‐induced structural phase transitions in MOFs composed of 1D rods include [In_3_(TTTA)_2_(OH)_3_(H_2_O)]·(DMA)_3_ (BUT‐70, TTTA^3−^ = (2*E*,2′*E*,2″*E*)‐3,3′,3″‐(2,4,6‐trimethylbenzene‐1,3,5‐triyl)‐triacrylate; DMA = N,N‐dimethylacetamide) [51], in which immersion in mixed solvents such as methanol (MeOH), DMA, or H_2_O induces a structural change in the In–O–C rods, resulting in a variation in gas adsorption capacity. Another example is Ni_2_L·8H_2_O (STA‐12, L = O_3_PCH_2_NC_4_H_8_NCH_2_PO_3_) [52], where adsorption/desorption of water molecules causes a structural change in the Ni–O rods. However, in these cases the dimensionality of the framework structure remains unchanged, and many of these transformations are irreversible [53, 54, 55, 56].

**FIGURE 1:**
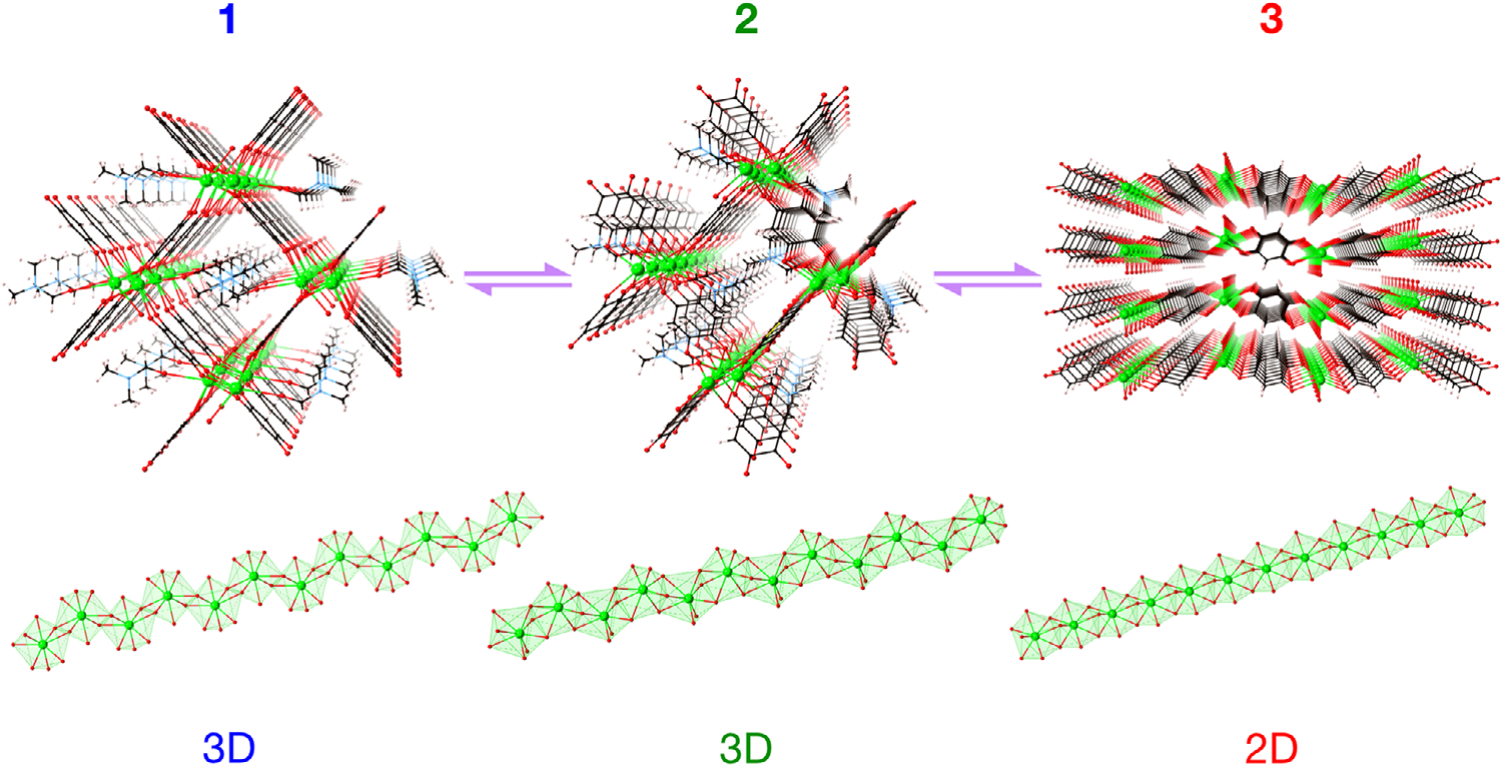
Overview of the discovery in this study. MOF **1**, a 3D framework constructed from rigid Pb–O rods, contains coordinative guest molecules (DMF and H_2_O) within its 1D pores. Exchange of these pore guests triggers reversible structural phase transitions, yielding **2** with a 3D framework and **3** with a 2D layered structure, accompanied by deformation of the Pb–O rods and a change in framework dimensionality.

Here, we report a reversible structural phase transition accompanied by a change in framework dimensionality, induced by coordinative‐guest‐molecule exchange, in a MOF composed of 1D Pb–O rods, [Pb(DHBQ)(DMF)(H_2_O)] (**1**, H_2_DHBQ = 2,5‐dihydroxy‐1,4‐benzoquinone). Compound **1** is a 3D MOF having 1D pores, in which Pb–O rods composed of Pb^2+^ ions and oxygen atoms from DHBQ^2−^ act as the secondary building units (SBUs). Within the pores, crystallization DMF and H_2_O molecules are present as coordinative guest molecules. Removal of the H_2_O molecules induces a structural transformation to [Pb(DHBQ)(μ‐DMF)] (**2**), which is a 3D MOF structure containing a 1D porous structure. Moreover, exchange of the coordinative DMF molecules located within the pores of **2** with H_2_O molecules results in a structural transformation to [Pb(DHBQ)(μ‐H_2_O)] (**3**), a MOF with a 2D layered structure where the Pb–O rods are bridged by DHBQ^2−^ ligands. Notably, all of these transformations proceed reversibly as summarized in Figure 1. To the best of our knowledge, **1** is the first example of a MOF containing rigid M–O rods as SBUs that exhibits a reversible structural phase transition accompanied by a change in framework dimensionality induced by guest‐molecule exchange.

## 2 Results and Discussion

### 2.1 Synthesis and Characterization of **1**

**FIGURE 2:**
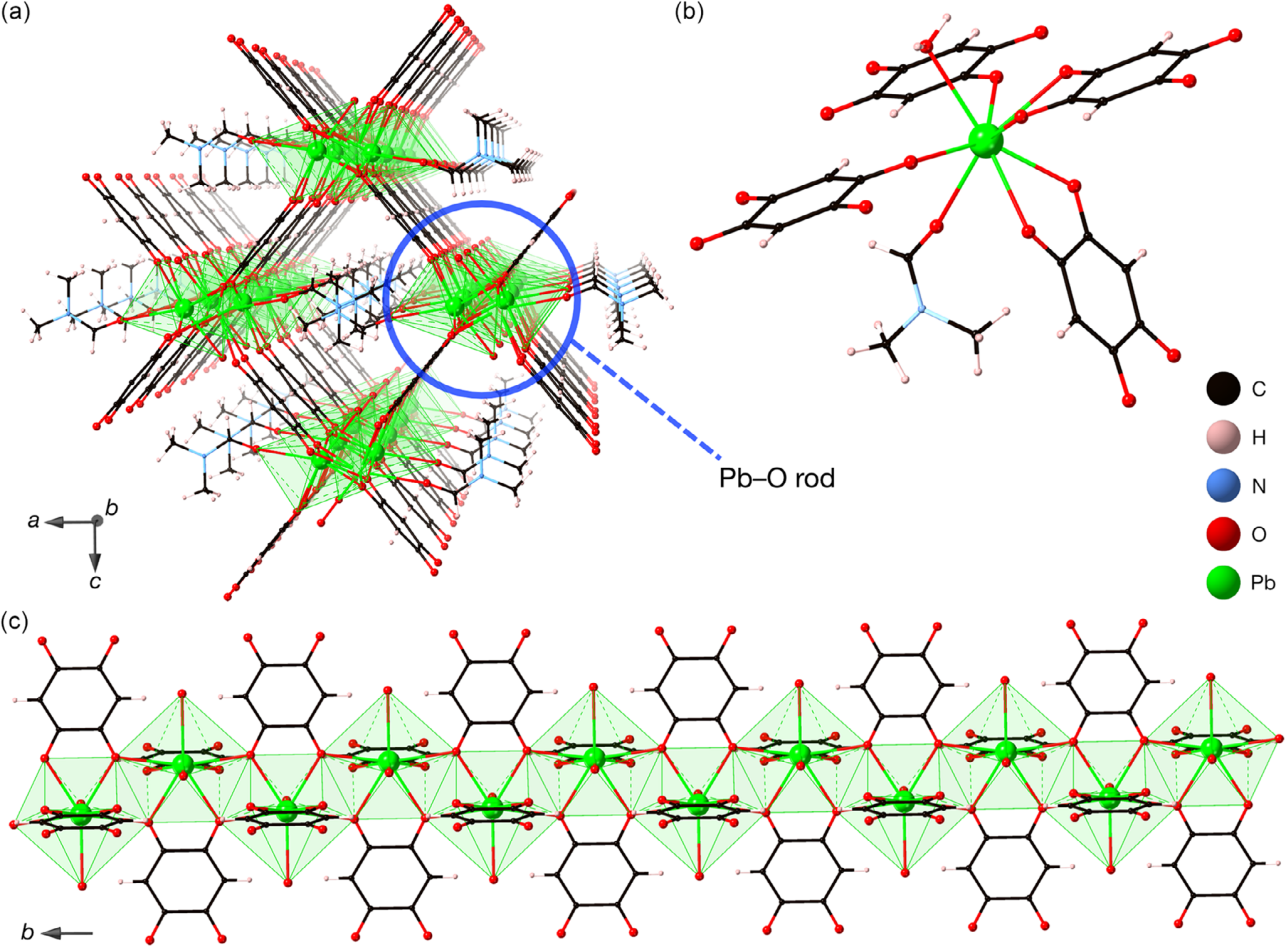
X‐ray crystal structure of **1** at 90 K. (a) 3D packing structure along the *b*‐axis, where Pb–O rod is highlighted by a purple circle. (b) Coordination geometry around the Pb^2+^ center. (c) Pb–O rod structure. All panels are drawn in a ball‐and‐stick model, and a polyhedral model is also used for the Pb–O rod. C, H, N, O, and Pb atoms are drawn in black, pink, blue, red, and green, respectively.

Dark red block‐shaped single crystals of [Pb(DHBQ)(DMF)(H_2_O)] (**1**) were successfully obtained by heating a mixture of lead nitrate and H_2_DHBQ in a DMF/H_2_O mixed solvent at 50°C (Figure S1a). Elemental analysis suggested that Pb, DHBQ^2−^, and the crystallization solvents DMF and H_2_O are present in a 1:1:0.1:1 ratio. As described later, single‐crystal X‐ray diffraction (SCXRD) measurements performed under conditions where the crystallization solvents were not lost confirmed that DMF and H_2_O are present in a 1:1 ratio in as‐synthesized **1**. Therefore, it is suggested that DMF molecules in **1** are readily removed under air‐drying conditions. The crystal structure of **1** was clearly determined by SCXRD. The crystal structure of **1** at 90 K is shown in Figure 2. A total of four DHBQ^2−^ units coordinate to one Pb ion. Among these, two DHBQ^2−^ coordinate through two oxygen atoms, while the other two DHBQ^2−^ ligands coordinate through one oxygen atom (Figure 2b). The latter DHBQ^2−^ ligands further coordinate to adjacent Pb ions to form bridging structures, thereby generating a 3D network structure (Figure 2c, Pb–O rod; see below). In addition, one DMF molecule and one H_2_O molecule, which serve as crystallization solvents, are each coordinated to the Pb ion as terminal ligands, and the coordinated H_2_O molecule interacts with the DHBQ^2−^ ligand through a hydrogen bond (Figures S2 and S3). Overall, since the ratio of Pb to DHBQ^2−^ is 1:1, the oxidation state of the Pb ion is +2, which is consistent with the results of the elemental analysis as described above.

On the other hand, in the 3D packing structure, the oxygen atoms of the bridging DHBQ^2−^ ligands connect adjacent Pb^2^^+^ ions, thereby forming infinite Pb–O rods along the *b*‐axis direction (Figure 2c). In addition, DHBQ^2−^ ligands bridge neighboring infinite Pb–O rods, resulting in the formation of a 1D porous structure (Figure 2a,c). The coordinating guest molecules DMF and H_2_O are exposed on the surfaces of these 1D pores. Focusing on the network motif within the Pb–O rods, the infinite Pb–O rods in **1** can be regarded as consisting of distorted PbO_8_ polyhedra connected in an edge‐sharing manner, where adjacent Pb^2+^ ions are bridged by two oxygen atoms derived from DHBQ^2−^ (Figure 2c). In the Pb–O rod structure, the nearest Pb^2+^−Pb^2+^ distance is 4.31 Å, while the Pb^2+^−O distances are 2.49 and 2.66 Å, respectively (Figure S4). In the diffuse reflectance spectrum of **1** (Figure S5), a broad absorption band straddling the visible and ultraviolet regions was observed, reflecting the reddish‐brown color of the crystals. Based on the results of time‐dependent density‐functional theory (TD‐DFT) calculation conducted using a model structure containing two Pb^2+^ centers derived from the single‐crystal X‐ray structure, the low‐energy absorption around 1.95 eV can be assigned to a ligand‐to‐metal charge transfer (LMCT) transition from the π orbital of the DHBQ^2−^ ligand to the sσ* orbital of Pb. Also, a broad absorption band spanning the visible to ultraviolet region can be attributed to intra/interligand electronic transitions involving π and π* orbitals, together with LMCT transitions (Figure S6, Tables S1 and S2).

### 2.2 Structural Transformation to a DMF‐Exchanged Phase With a Change in the Rod Structure

**FIGURE 3:**
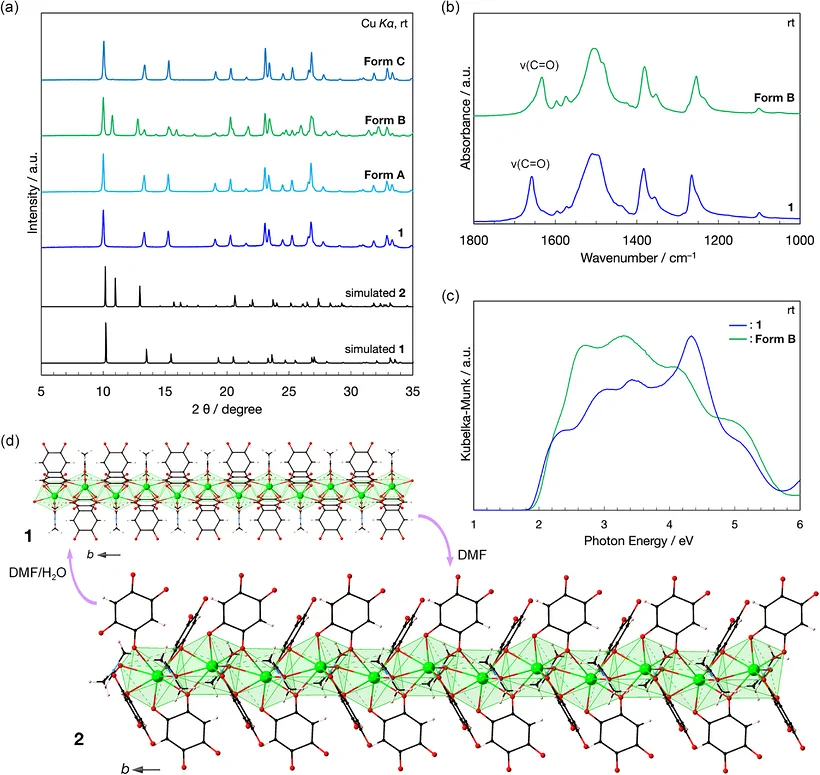
Structural transformation from **1** to DMF‐exchanged form. (a) PXRD patterns in different states at rt. Simulated patterns of **1** and **2** are also shown. (b) FTIR spectra in different states at rt. (c) Diffuse reflectance spectra in different states at rt. (d) Comparison of Pb–O rod structure between **1** and **2**. Color code as in Figure 2.

As described above, as‐synthesized **1**, DMF, and H_2_O molecules coordinated to Pb^2^^+^ ions are present within the 1D pores as coordinating guest molecules. We investigated the exchange of guest molecules by immersing the polycrystalline powder of **1** in various solvents and observed an intriguing structural transformation. When the polycrystalline powder of as‐synthesized **1** was briefly immersed in methanol to obtain a starting sample (**Form A**, see Supporting Information for details) and subsequently kept in pure DMF at room temperature (rt) for 3 days, polycrystalline powder with a different crystal structure (**Form B**: DMF‐exchanged form of **Form A**) was obtained. The powder X‐ray diffraction (PXRD) patterns of each sample are shown in Figure 3a. The PXRD pattern of **Form A** agrees well with that of as‐synthesized **1**, as well as with the simulated pattern based on the single‐crystal X‐ray structure of **1**, indicating that **Form A** is isostructural with **1**. In contrast, **Form B**, obtained after standing in pure DMF, was found to possess a crystal structure completely different from that of **1**. Interestingly, when **Form B** was kept at rt for 3 days in a DMF/H_2_O mixed solvent, which is similar to the synthetic condition of **1,** it reversibly returned to the same structure as **1** (**Form C**: DMF/H_2_O‐exchanged form of **Form B**, see Figure 3a). These results suggest that structural transformation in **1** occurs through the exchange of coordinative guest molecules within the pores. In the Fourier transform infrared (FTIR) spectra (Figure 3b), the ν(C = O) stretching modes of coordinated DMF were observed at 1658 and 1633 cm^−1^ for **1** and **Form B** (identical to **2**, see below), respectively, both appearing at lower wavenumbers than that of free DMF (∼1662 cm^−1^) [57]. As discussed below, the coordination modes of DMF differ between **1** and **Form B**, adopting terminal and bridging configurations, respectively. Reflecting this difference, the ν(C = O) mode for **Form B**, where DMF adopts a bridging coordination mode, is shifted to a lower wavenumber relative to that of **1** with terminally coordinated DMF as seen in Figure 3b. Furthermore, the diffuse reflectance spectra revealed that **Form B** exhibited absorption bands different from those of **1**, implying a change in the electronic states derived from guest exchange (Figure 3c).

**FIGURE 4:**
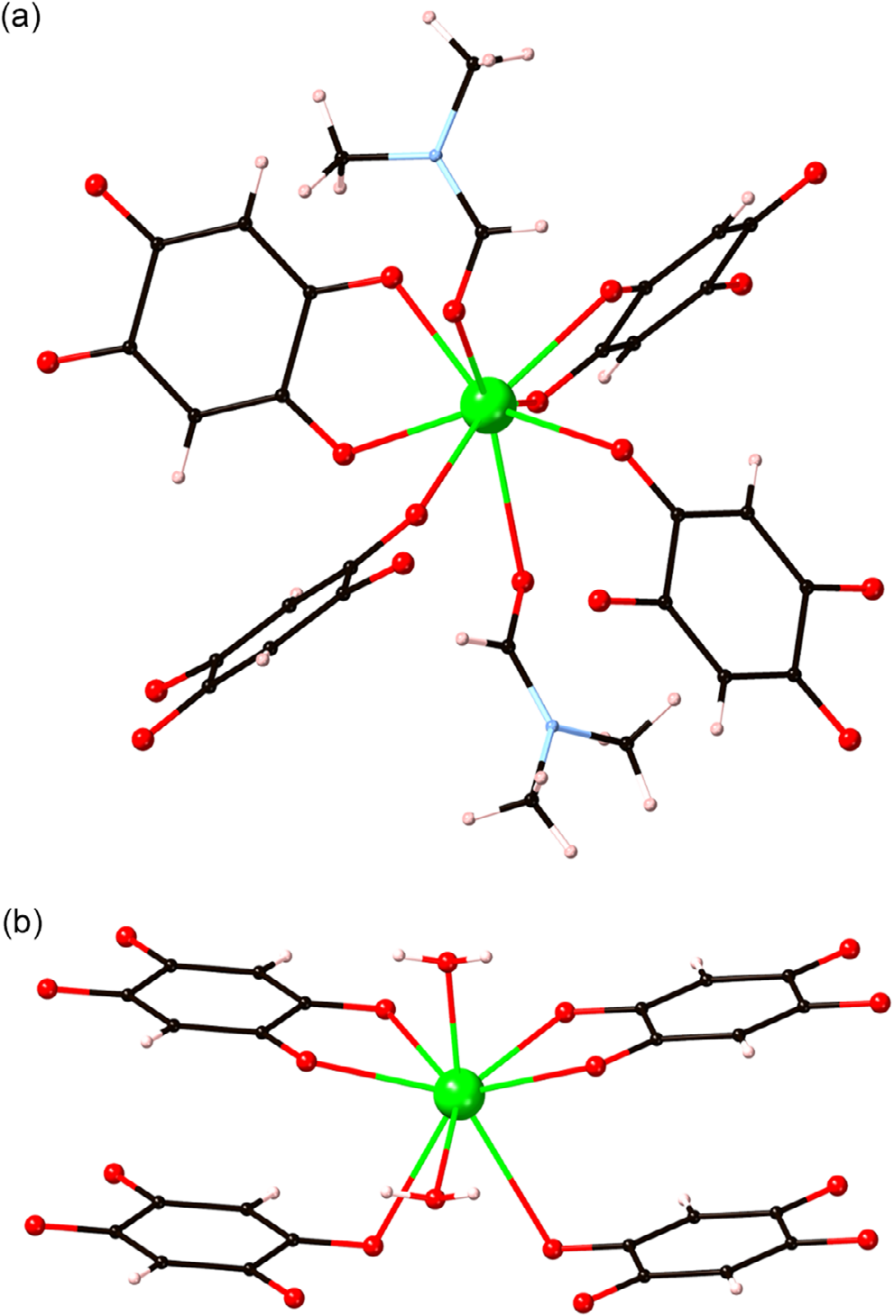
Coordination geometries around Pb^2+^ center in (a) **2** and in (b) **3**. Color code as in Figure 2.

Although structural analysis of **Form B** could not be achieved through guest exchange using polycrystalline powder, the use of pure DMF as the solvent enabled us to successfully obtain dark red block‐shaped single crystals of [Pb(DHBQ)(μ‐DMF)] (**2**), in which only the DMF molecule presents as a coordinative guest molecule (Figure S1b). The crystal structure of **2** (Pb–O rod structure) determined by SCXRD analysis is shown in Figure 3d. The coordination geometry around the Pb^2+^ center of **2** is also shown in Figure 4a. In **2**, the H_2_O molecules coordinated to Pb^2+^ observed in **1** are absent, and only coordinative DMF molecules are present as crystallization solvent molecules. This result is consistent with the elemental analysis, which indicates a Pb:DHBQ^2−^:DMF ratio of 1:1:1. It was revealed that **2** possesses a Pb–O rod structure different from that of **1**. In **2**, two DHBQ^2−^ ligands and one oxygen atom from a DMF molecule bridge adjacent Pb^2+^ ions, forming infinite Pb–O rods extending along the *b*‐axis. As will be discussed later, a notable feature of the structural transformation from **1** to **2** is the change in the coordination mode of the coordinative guest molecule (DMF) from terminal coordination to bridging coordination (Figure S7). In addition, DHBQ^2−^ ligands connect neighboring Pb–O rods, resulting in a 3D framework with 1D pores. The simulated PXRD pattern based on the single‐crystal X‐ray structure of **2** agrees well with the PXRD pattern of **Form B** obtained as a polycrystalline powder, confirming that **Form B** obtained through guest exchange of the polycrystalline powder corresponds to the DMF‐exchanged form. A detailed comparison of the Pb–O rod structures in **1** and **2** (Figure 3d) reveals notable differences. In the Pb–O rods of **1**, adjacent Pb^2+^ ions are bridged by two oxygen atoms, whereas in the Pb–O rods of **2**, the Pb^2+^−Pb^2+^ pairs are bridged by three oxygen atoms. In addition, in **1**, one DHBQ^2−^ molecule bridges three neighboring Pb^2+^ ions, while in **2**, one DHBQ^2−^ molecule bridges two adjacent Pb^2+^ ions. Thus, it was demonstrated that exchanging all coordinative guest molecules in **1** with DMF induces a structural change in the Pb–O rod. In **2**, the Pb^2+^−Pb^2+^ distance is 4.00 Å, which is approximately 0.3 Å shorter than that in **1** (Figure S8). This rod structure transformation involving a change in the coordination mode of the DMF molecule discussed above provides different absorption bands from those of **1**. In particular, on the basis of the X‐ray crystallographic analyses and TD‐DFT calculations of the electronic transitions, the change in the coordination environment around the Pb^2+^ centers accompanying the phase transition from **1** to **2** is reflected in the optical absorption at around 2 eV in the diffuse reflectance spectra. The lowest‐energy absorption bands in **1** and **2** are assigned to electronic transitions from ligand‐based π orbital to Pb^2+^ sσ* orbital. In contrast, for **2**, the lowest‐energy absorption is more specifically attributed to a transition from ligand pπ (O) orbital to Pb^2+^ sσ* orbital, accompanied by a pronounced increase in oscillator strength. This marked enhancement indicates a substantial change in the electronic structure induced by the change in the coordination mode (Figure S9, Tables S3 and S4).

### 2.3 Structural Transformation to an H_2_O‐Exchanged Form With a Change in Framework Dimensionality

**FIGURE 5:**
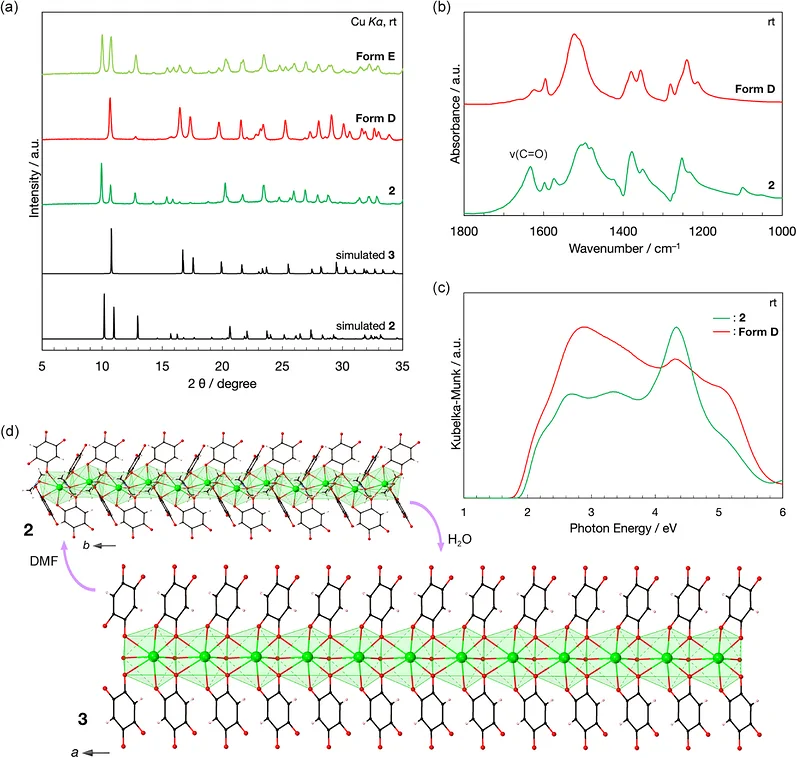
Structural transformation from **2** to H_2_O‐exchanged form. (a) PXRD patterns in different states at rt. Simulated patterns of **2** and **3** are also shown. (b) FTIR spectra in different states at rt. (c) Diffuse reflectance spectra in different states at rt. (d) Comparison of Pb–O rod structure between **2** and **3**. Color code as in Figure 2.

Next, we investigated the exchange of the coordinative guest molecule (DMF) in **2**, and observed a reversible structural transformation accompanied by a change in the structural dimensionality of the overall framework. When the polycrystalline powder of **2** was kept in pure H_2_O at rt for 5 days, a phase having a different crystal structure (**Form D**: H_2_O‐exchanged form of **2**) was obtained (Figure 5a). In contrast, when the polycrystalline powder of **Form D** was kept in pure DMF at rt for 3 days, a reversible transformation back to a crystal structure identical to that of **2** was observed (**Form E**: DMF‐exchanged form of **Form D**, see Figure 5a). In the FTIR spectra shown in Figure 5b, the ν(C = O) mode of DMF observed in **2** disappeared in **Form D**, suggesting that in **Form D**, the coordinative DMF molecules present in **2** have been replaced by H_2_O molecules. Furthermore, the diffuse reflectance spectra revealed that **Form D** exhibits an absorption band clearly different from that of **2** (Figure 5c).

**FIGURE 6:**
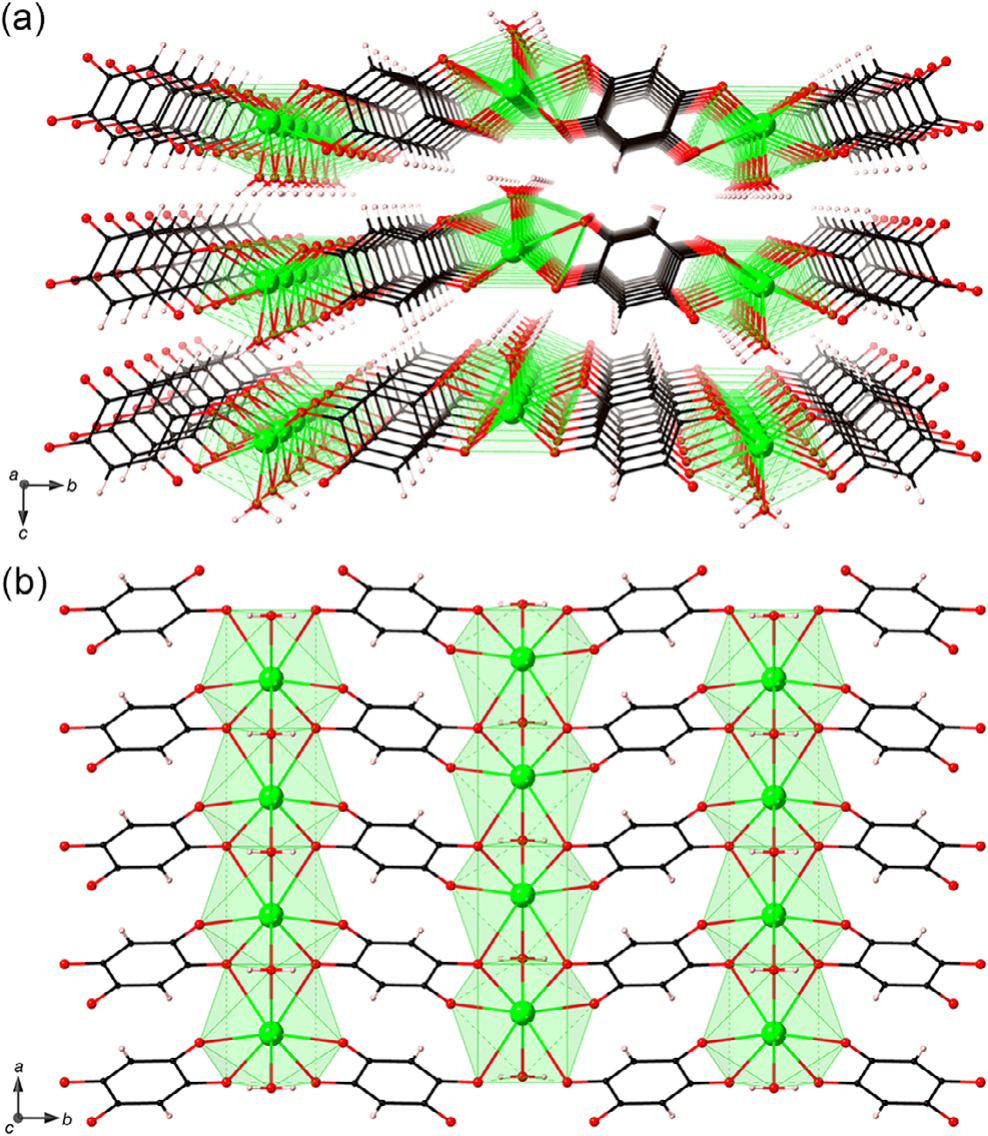
2D layered structure of **3**. 3D packing structure along the *a*‐axis (a) and *c*‐axis (b).

We also attempted to obtain the H_2_O‐exchanged form by direct synthesis. By conducting the reaction at 5°C using pure H_2_O as the solvent, we successfully obtained dark red block‐shaped single crystals of [Pb(DHBQ)(μ‐H_2_O)] (**3**), in which only H_2_O molecules are present as coordinative guest molecules (Figure S1c). The simulated PXRD pattern of **3**, based on SCXRD analysis, shown later, agrees well with that of **Form D**. In addition, elemental analysis of **3** indicates a Pb:DHBQ:H_2_O ratio of 1:1:1. These results confirm that the guest exchange using the polycrystalline powder of **2** produced the H_2_O‐exchanged form. The single crystal X‐ray structure of **3** is shown in Figures 5d and 6. The coordination geometry around the Pb^2+^ center of **3** is also shown in Figure 4b. Notably, although this compound possesses a rigid Pb–O rod structure, exchange of the coordinative guest molecules induces a change in the framework dimensionality. In **3**, two DHBQ^2−^ ligands and the oxygen atom of one H_2_O molecule bridge adjacent Pb^2+^ ions, forming infinite Pb–O rod structures extending along the *a*‐axis. Note that, similar to **2**, the coordinative guest molecule (H_2_O) in **3** also adopts a bridging coordination mode (Figure S10). In addition, DHBQ^2−^ ligands connect neighboring Pb–O rods.

In **1** and **2**, the connections between adjacent Pb–O rods via DHBQ^2−^ extend in 2D, resulting in an overall 3D framework. In contrast, in **3**, the connections between adjacent Pb–O rods are restricted to only the *b*‐axis direction, leading to the formation of an overall 2D layered structure (Figure 6). The Coordinatives H_2_O molecule discussed above further interacts with DHBQ^2−^ ligand of the neighboring 2D layer, through the interlayer hydrogen bonding (Figure S11). Next, comparing the Pb–O rod structures of **2** and **3**, it is found that in **3,** as in **2**, adjacent Pb^2+^ ions are bridged by three oxygen atoms. However, while in **2** the DHBQ^2−^ ligands alternately bridge two adjacent Pb^2+^ ions (along the 2_1_ screw axis), in **3** they bridge in the same direction, suggesting that this difference leads to the change in structural dimensionality. Furthermore, accompanying the structural transformation from **2** to **3**, the Pb^2+^−Pb^2+^ distance decreases from 4.00 Å to 3.86 Å (Figure S12). Another important feature of **3**, which is not observed in the structures of **1** or **2**, is the presence of pronounced π–π interactions between adjacent DHBQ^2−^ ligands within the Pb–O rods (Figure S13). These results suggest that, during the phase transition from **2** to **3**, the coordinative DMF molecules are entirely replaced by H_2_O, which is smaller than DMF, thereby allowing π–π interactions to contribute to the stabilization of the overall structure. This π–π interaction within the Pb–O rod provided a drastic change in the diffuse reflectance spectrum, where strong intermolecular π–π* transitions of adjacent DHBQ^2−^ ligands appear at around 2.9 eV as confirmed by TD‐DFT calculations. Moreover, the optical absorption at around 2 eV, assigned to an electronic transition from ligand pσ orbitals to Pb^2^^+^ sσ* orbitals, exhibited a pronounced increase in oscillator strength upon the phase transition from **2** to **3**. This marked enhancement suggests a change in the electronic structure induced by the alteration in the coordination mode as observed in the transition from **1** to **2** (Figures 5c, S14, and S15, Tables S5 and S6).

We also examined the thermal stability of **1–3** together with the removal of the coordinative guest molecules (Figures S16–S18). Removal of the coordinative guest molecules (DMF and H_2_O) from **1** and **2** resulted in the formation of the same desolvated (activated) phase possessing an identical crystal structure as confirmed by thermogravimetric analysis (TGA) and PXRD (Figure S19). N_2_ adsorption isotherm measurements at 77 K indicated that this desolvated form is a nonporous framework (Figure S20). In contrast, the removal of the coordinated H_2_O molecules in **3** was suggested to proceed concomitantly with decomposition of the crystal structure, and the PXRD pattern of the activated phase confirmed the transformation into a mixture of multiple crystalline phases (Figure S20).

### 2.4 Structural Transformation Triggered by Coordinative‐Guest Exchange

In this study, we observed a pronounced structural phase transition in **1** triggered by the exchange of coordinative guest molecules located within its pores. This structural transformation is accompanied by substantial changes in the Pb–O rod units as well as in the overall dimensionality of the framework. A comparison of the coordination environments around the Pb^2+^ centers in **1–3** reveals a marked difference in the coordination geometry of the coordinative guest molecules. SCXRD study shows that, in **1**, the coordinative solvent molecules DMF and H_2_O coordinate to the Pb^2+^ center as terminal ligands (Figure S2). In contrast, in **2** and **3**, DMF or H_2_O molecules adopt bridging coordination modes, linking two Pb^2+^ centers (Figures S7 and S10). Such a change in the coordination geometry of the guest molecules is likely responsible for the rearrangement of the Pb–O rod units, ultimately leading to the observed change in the framework dimensionality.

On the basis of the experimental observations described above, the guest‐induced structural transformations can be rationalized as follows. In **1**, which was synthesized in a DMF/H_2_O mixed solvent, both DMF and H_2_O molecules competitively coordinate to the Pb^2+^ centers, resulting in the coexistence of both species as terminal ligands. In this framework, **1**, the coordinated DMF molecules are partially released upon air‐drying, as confirmed by elemental analysis. When **1** is immersed in pure DMF, the removal of coordinated H_2_O is promoted from the viewpoint of chemical equilibrium, leading to the preferential coordination of DMF molecules to the Pb^2+^ centers. In this situation, DMF can adopt a bridging coordination mode linking two Pb^2+^ ions, which is energetically more favorable than terminal coordination as in **1**. This change in the coordination mode is considered to drive the structural transformation from **1** to **2**, accompanied by a rearrangement of the Pb–O rod structure. Consistent with this interpretation, the coordinated DMF molecules in **2** remain bound even after air‐drying as evidenced by the elemental analysis.

Moreover, when **2** is placed in pure H_2_O, the guest DMF molecules are replaced by H_2_O to yield **3**. The dissociation of bridging DMF ligands is favored under these conditions, and the smaller and more strongly coordinating H_2_O molecules adopt bridging coordination between Pb^2+^ centers, resulting in shorter Pb^2+^−Pb^2+^ separations within the Pb–O rods. Owing to the smaller molecular size of H_2_O, the connectivity between adjacent Pb–O rods can reorganize, accompanied by a rearrangement of DHBQ^2−^ ligand, which allows additional stabilization through π–π interactions between ligands (Figure S21). This structural reorganization ultimately leads to a change in the framework dimensionality during the transformation from **2** to **3**.

## 3 Conclusion

In conclusion, we successfully synthesized a MOF, **1**, representing the first example of a MOF that exhibits reversible transformations of both the Pb–O rod structure and the dimensionality of the framework triggered by the exchange of coordinative guest molecules. The coordinative guest DMF and H_2_O molecules trapped in the 1D pores of **1** can be reversibly exchanged, leading to successive structural phase transitions to distinct frameworks, **2** and **3**, as clearly revealed by X‐ray diffraction studies. Notably, these transformations of the Pb–O rod structure and the overall framework dimensionality are accompanied by corresponding changes in the electronic states, as confirmed by spectroscopic measurements and TD‐DFT calculations. The results presented here demonstrate that guest coordination can serve as an effective trigger for controllable and reversible structural transformations in MOFs. Porous materials featuring moderately flexible metal–oxo rod motifs derived from post‐transition‐metal ions, as demonstrated in this study, hold considerable promise for application uses that exploit their structural flexibility. In particular, such systems may enable guest‐induced switching of properties—such as magnetism and electrical conductivity—thereby offering potential use as sensor materials, switching devices, and drug delivery systems.

## 4 Experimental Section

**General**: All reagents and solvents were purchased from Kanto Chemical and Tokyo Chemical Industry and were used without further purification. Fourier‐transform infrared (FTIR) spectrum was measured with a JASCO FT/IR‐4x spectrometer at rt. Powder X‐ray diffraction (PXRD) pattern was collected using Rigaku MiniFlex600‐C X‐ray diffractometer (Cu *K*α radiation) at rt. Diffuse reflectance spectrum diluted in CaF_2_ powder was recorded using a JASCO V‐570 spectrometer with a ISN‐470 60 mm ϕ integrating sphere apparatus at rt. Thermogravimetric analysis (TGA) was performed with a Rigaku Thermo plus EVO2 at a heating rate of 5 K/min in a constant flow of N_2_ gas. N_2_ sorption measurement was performed on BELSORP‐max apparatus (MicrotracBEL, Japan, Corp.). The polycrystalline powder was dried under high vacuum (< 10^−1^ Pa) at 200°C for 3 h prior to sorption measurement. Elemental analysis was performed using MT‐6 CHN recorder (Yanaco) at the Organic Microanalysis Laboratory, Kyoto University.

**Synthesis of [Pb(DHBQ)(DMF)(H_2_O)] (1)**: Pb(NO_3_)_2_ (0.50 mmol, 165.8 mg) was dissolved in a DMF/H_2_O mixed solvent (4:1, 5 mL). H_2_DHBQ (1.5 mmol, 210.8 mg) was dissolved in 5 mL of DMF. These solutions were mixed and heated in a 20 mL screw capped vial at 50°C for 96 h. Dark red block‐shaped single crystals of **1** were obtained (Figure S1a). Elemental analysis (%) calcd for Pb(C_6_H_2_O_4_)(C_3_H_7_NO)_0.07_(H_2_O): C 20.25, H 1.23, N 0.26; found: C 20.13, H 1.36, N 0.34.

**Synthesis of [Pb(DHBQ)(μ‐DMF)] (2)**: Single crystals of **2** were obtained from similar to the preparation of **1**, but Pb(NO_3_)_2_ was dissolved in 5 mL of pure DMF. Dark red block‐shaped single crystals of Pb(DHBQ)(DMF) (**2**) were obtained (Figure S1b). Elemental analysis (%) calcd for Pb(C_6_H_2_O_4_)(C_3_H_7_NO): C 25.84, H 2.17, N 3.35; found: C 25.66, H 2.23, N 3.37.

**Synthesis of [Pb(DHBQ)(μ‐H_2_O)] (3)**: Pb(NO_3_)_2_ (0.030 mmol, 10.0 mg) and H_2_DHBQ (0.030 mmol, 4.25 mg) were separately dissolved in 5 mL of H_2_O. These solutions were mixed and kept in a 20 mL screw capped vial at 5°C for 64 h. Dark red block‐shaped single crystals of **3** were obtained (Figure S1c). Elemental analysis (%) calcd for Pb(C_6_H_2_O_4_)(H_2_O): C 19.84, H 1.11; found: C 19.84, H 1.19. Note: A compound obtained from the reaction of Pb(NO_3_)_2_ and H_2_DHBQ in H_2_O has been reported previously; however, only elemental analysis data was reported and its crystal structure and detailed chemical composition have not been clarified [58].

### Guest exchange procedures

**1 to DMF‐exchanged form**: Polycrystalline powder of **1** was rinsed with MeOH for 1 min (**Form A**), which prompts smooth guest exchange. Then, the sample was immersed in pure DMF at rt for 3 days, to give DMF‐exchanged form (**Form B**) as brown microcrystalline powder. The crystal structure of **Form B** is identical to **2**, as confirmed by PXRD.

**DMF‐exchanged form to 1**: Polycrystalline powder of **Form B** was immersed in a DMF/H_2_O (4:1) mixture at rt for 3 days, which gave polycrystalline powder of **Form C** identical to **1**.

**2 to H_2_O‐exchanged form**: Polycrystalline powder of **2** was immersed in pure H_2_O at rt for 5 days, to give H_2_O‐exchanged form (**Form D**) as brown polycrystalline powder. The crystal structure of **Form D** is identical to **3**, as confirmed by PXRD.

**H_2_O‐exchanged form to 2**: Polycrystalline powder of **Form D** was immersed in pure DMF at rt for 3 days, which gave polycrystalline powder of **Form E** identical to **2**.

**Single‐crystal X‐ray diffraction (SCXRD)**: Single‐crystal XRD measurements were carried out using a Bruker D8 QUEST PHOTON III detector with graphite‐monochromated Mo *K*α radiation (*λ* = 0.71073 Å) at 90 K. The single crystal was mounted on MicroMesh (MiTeGen) with Paraton‐N oil (Hampton Research) to avoid the loss of guest molecules. The structures were solved by dual‐space method (SHELXT version 2018/2) [59] and refined by full‐matrix least‐squares refinement on *F* ^2^ (SHELXL version 2019/3) [60] using the CrystalStructure 4.3.2. software [61].

**Crystallographic data for 1**: *M*r = 436.39, crystal dimensions 0.10 × 0.10 × 0.08 mm^3^, orthorhombic, space group *Pnma* (no. 62), *a* = 11.5222(5) Å, *b* = 7.6274(3) Å, *c* = 13.1132(6) Å, *V* = 1152.45(9) Å^3^, Z = 4, *d*_calc_ = 2.515 g cm^−3^, *F*(000) = 808.0, *μ* = 14.681 mm^−1^, *T* = 90 K, A total of 16019 reflections were collected, of which 1097 reflections were unique (*R*_int_ = 0.0656). Refinement of 91 parameters led to the final *R*_1_ = 0.0279 (*I* > 2σ(*I*)), *wR*_2_ = 0.0748 (all data), GOF = 1.161, and residual electron density max/min = 1.14/–2.03 eÅ^−3^. CCDC reference number 2537129.

**Crystallographic data for 2**: *M*r = 418.37, crystal dimensions 0.07 × 0.03 × 0.03 mm^3^, monoclinic, space group *P*2_1_/*n* (no. 14), *a* = 10.5994(5) Å, *b* = 7.4074(4) Å, *c* = 13.7027(7) Å, *β* = 94.514(2), *V* = 1072.52(9) Å^3^, Z = 4, *d*_calc_ = 2.591 g cm^−3^, *F*(000) = 768.0, *μ* = 15.762 mm^−1^, *T* = 90 K, A total of 10629 reflections were collected, of which 1846 reflections were unique (*R*_int_ = 0.0956). Refinement of 145 parameters led to the final *R*_1_ = 0.0391 (*I* > 2σ(*I*)), *wR*_2_ = 0.0976 (all data), GOF = 1.061, and residual electron density max/min = 1.64/–1.43 eÅ^−3^. CCDC reference number 2537130.

**Crystallographic data for 3**: *M*r = 363.29, crystal dimensions 0.04 × 0.008 × 0.005 mm^3^, monoclinic, space group *P*2_1_/*m* (no. 11), *a* = 3.8603(2) Å, *b* = 16.428(1) Å, *c* = 5.3041(4) Å, *β* = 91.593(3), *V* = 336.24(4) Å^3^, Z = 2, *d*_calc_ = 3.588 g cm^−3^, *F*(000) = 324.0, *μ* = 25.105 mm^−1^, *T* = 90 K, A total of 6287 reflections were collected, of which 829 reflections were unique (*R*_int_ = 0.0834). Refinement of 49 parameters led to the final *R*_1_ = 0.0269 (*I* > 2σ(*I*)), *wR*_2_ = 0.0611 (all data), GOF = 1.083, and residual electron density max/min = 1.44/–1.74 eÅ^−3^. CCDC reference number 2537131.

**Computational study**: TD‐DFT calculations on electronic transition were performed using the Gaussian09W package [62], and the PBE1PBE functional [63]. A total of 400 states of electronic transition were considered. The SDD [64] (for model structure based on **1**) and LANL2DZ [65] (for model structures based on **2** and **3**) basis sets were used for Pb, and the 6–31G(d,p) [66] basis set was used for others. For these calculations, simplified model structures containing a single Pb–Pb dimer obtained from the Pb–O rod structure in single‐crystal structures of **1–3** were used. For details, see Supporting Information.

## Conflicts of Interest

The authors declare no conflicts of interest.

## Supporting information




**Supporting File**: chem71203‐sup‐0001‐SuppMat.pdf.

## Data Availability

The data that support the findings of this study are available from the corresponding author upon reasonable request.
