# Supplementary material for: Coordinative‐Guest‐Triggered Dimensional Crossover in a Metal–Organic Framework With Rigid Metal–Oxo Rods
Source: Chemistry. 2026 Jun 1;32(35):e71203. doi: 10.1002/chem.71203 (PMC13587648; doi:10.1002/chem.71203)
Supplement: Supplementary file 1 [file CHEM-32-e71203-s001.pdf]

Supporting Information

Coordinative-Guest-Triggered Dimensional Crossover in a Metal–Organic Framework with Rigid Metal–Oxo Rods

Keisuke Ishikawa,[1] and Kazuya Otsubo*[,1,2]

[1]Department of Chemistry, Faculty of Science, Tokyo University of Science, Shinjuku-ku, Tokyo, 162-8601, Japan

[2]Research Institute for Science and Technology, Division of Joint Research of Geometry and Natural Science, Tokyo University of Science, Chiba, 278-8510, Japan

*e-mail: otsubok@rs.tus.ac.jp

Table of Contents

Figures S1–S21 and Tables S1–S6 .... **pp.S2–S27**

Figures S1–S21 and Tables S1–S6

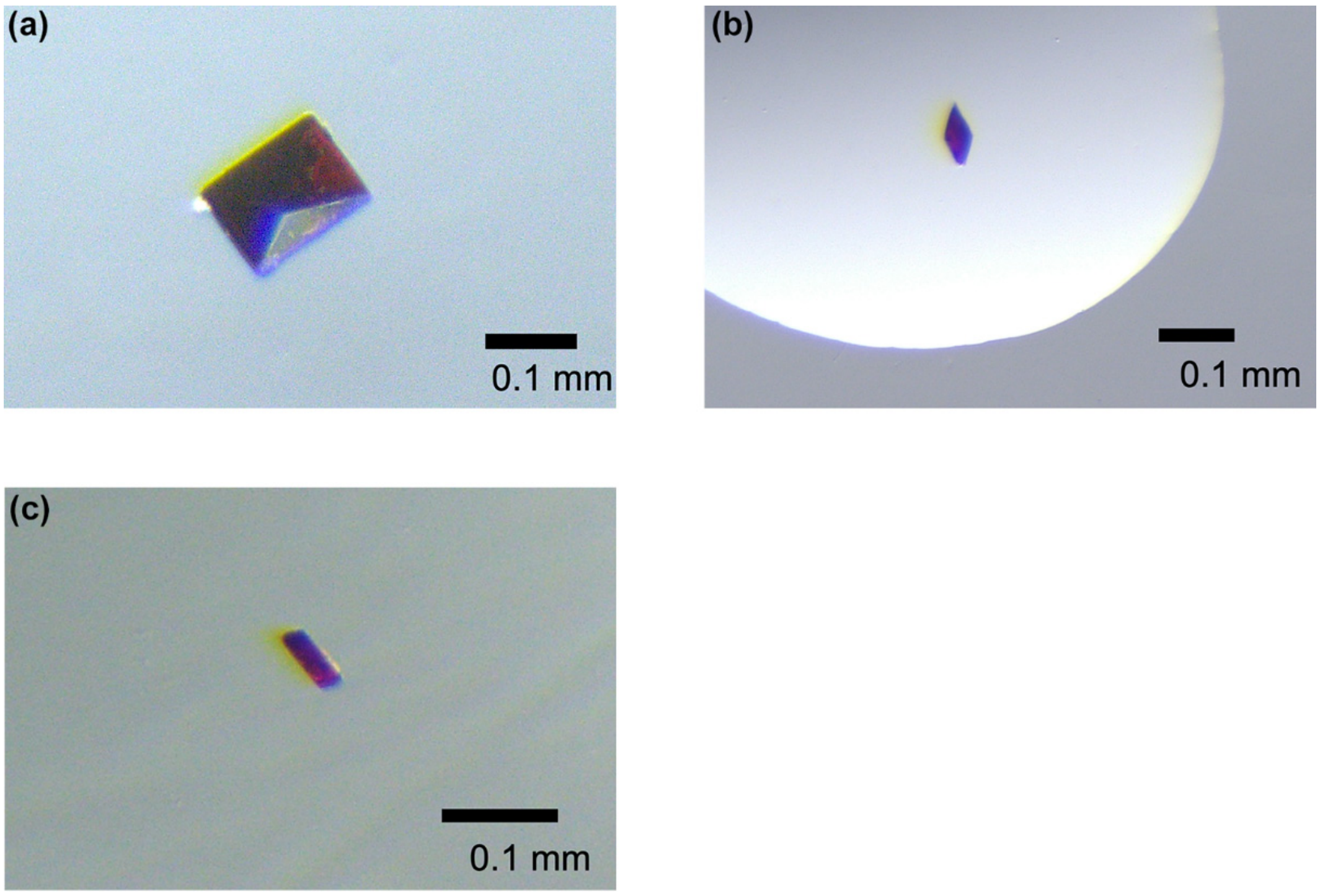

Figure S1. Typical examples of single crystal of **1** (a), **2** (b), and **3** (c).

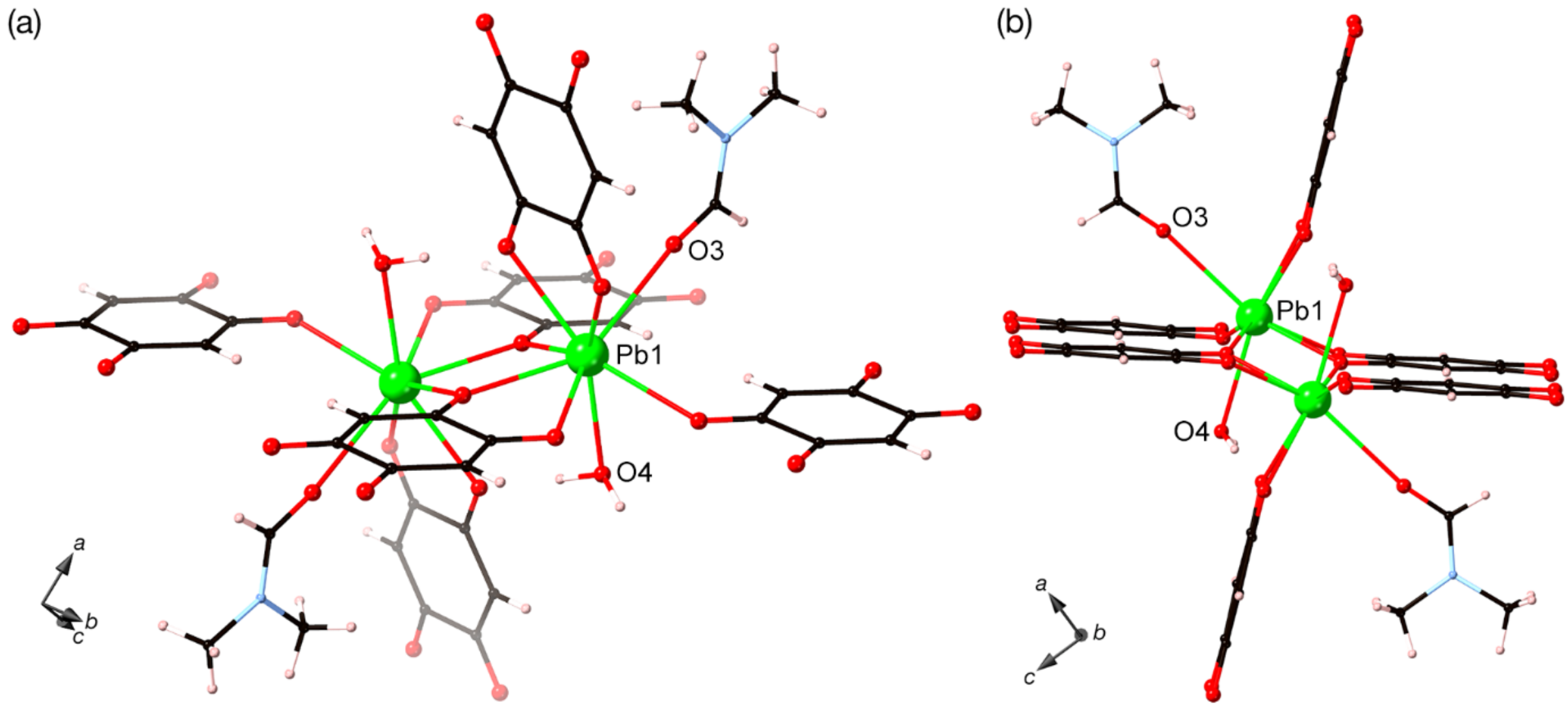

Figure S2. Coordination modes of DMF and H_2O molecules in **1** around Pb^{2+} center in different view. Color code as in Figure 2. Pb1–O3 and Pb1–O4 distances are 2.79, and 2.65 Å, respectively.

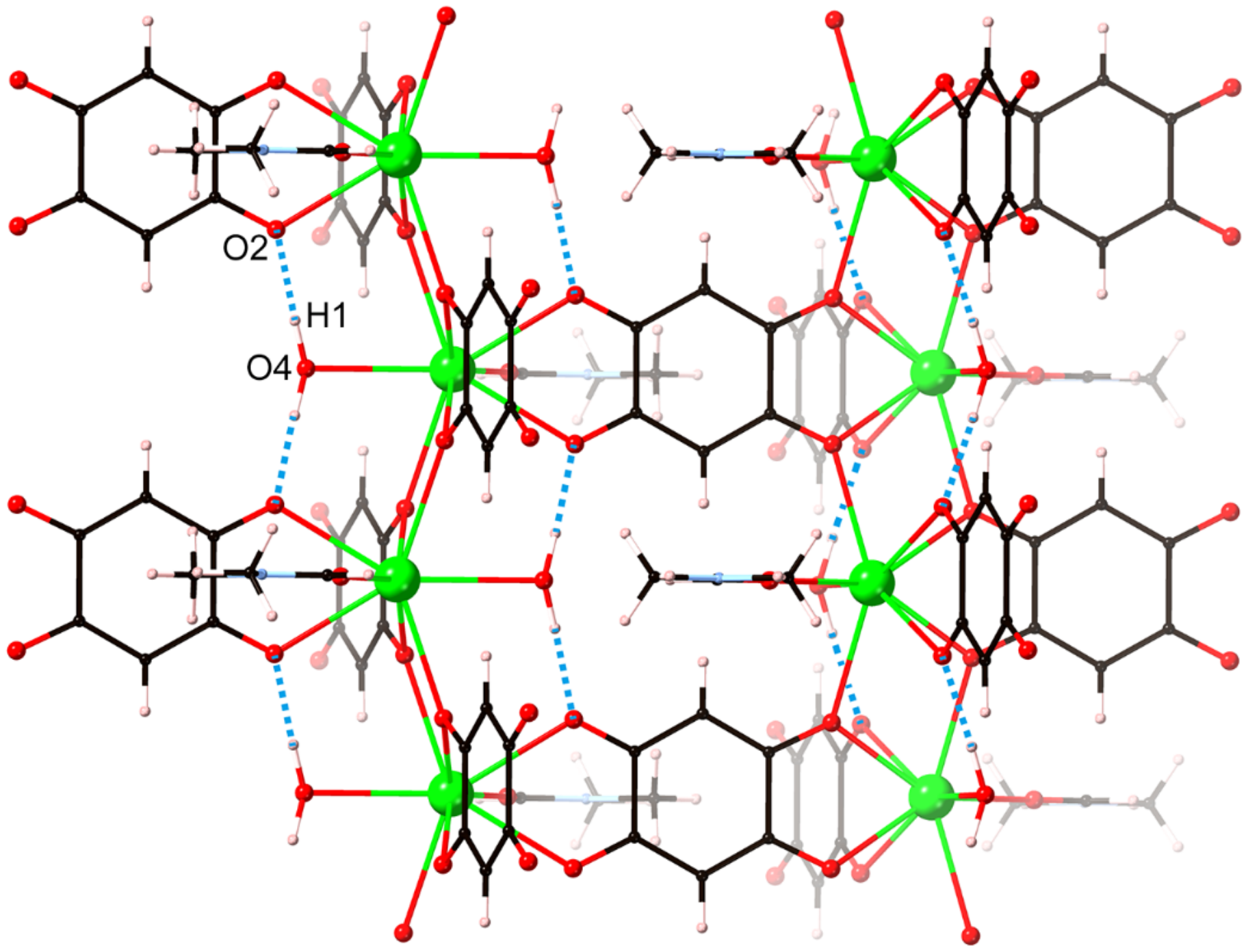

Figure S3. Hydrogen bonding in **1** between H_2O and $DHBQ^{2-}$ ligand. Color code as in Figure 2. O2–O4 distance is 2.79 Å.

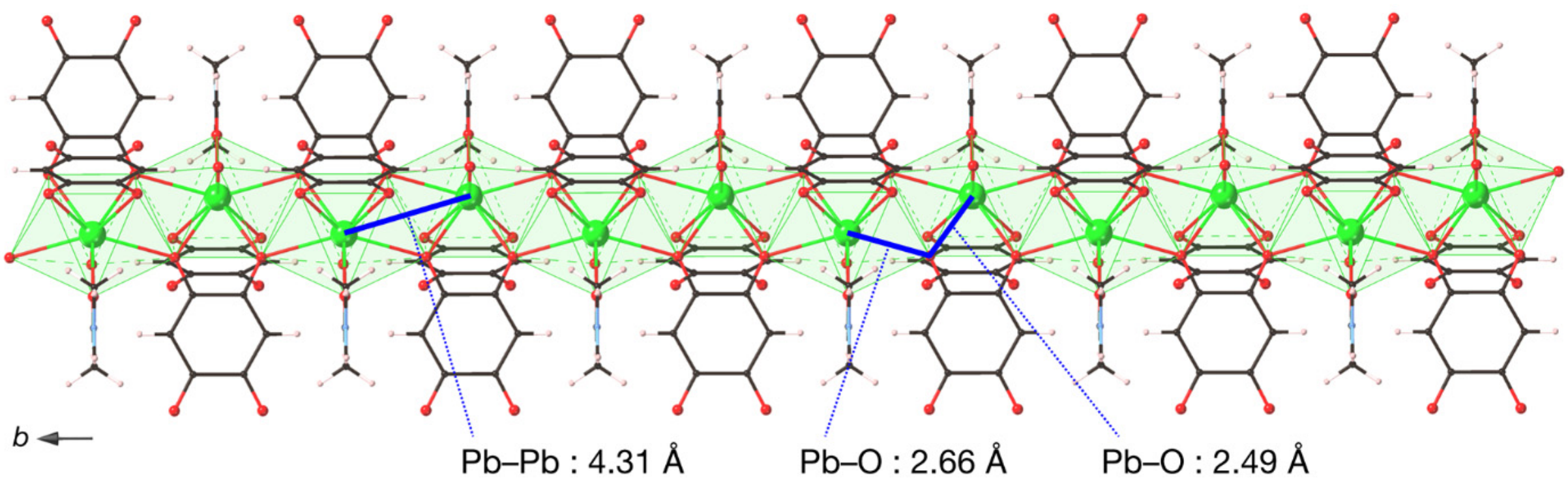

Figure S4. Pb–O structure of **1** (same as in Figure 2c). Color code as in Figure 2.

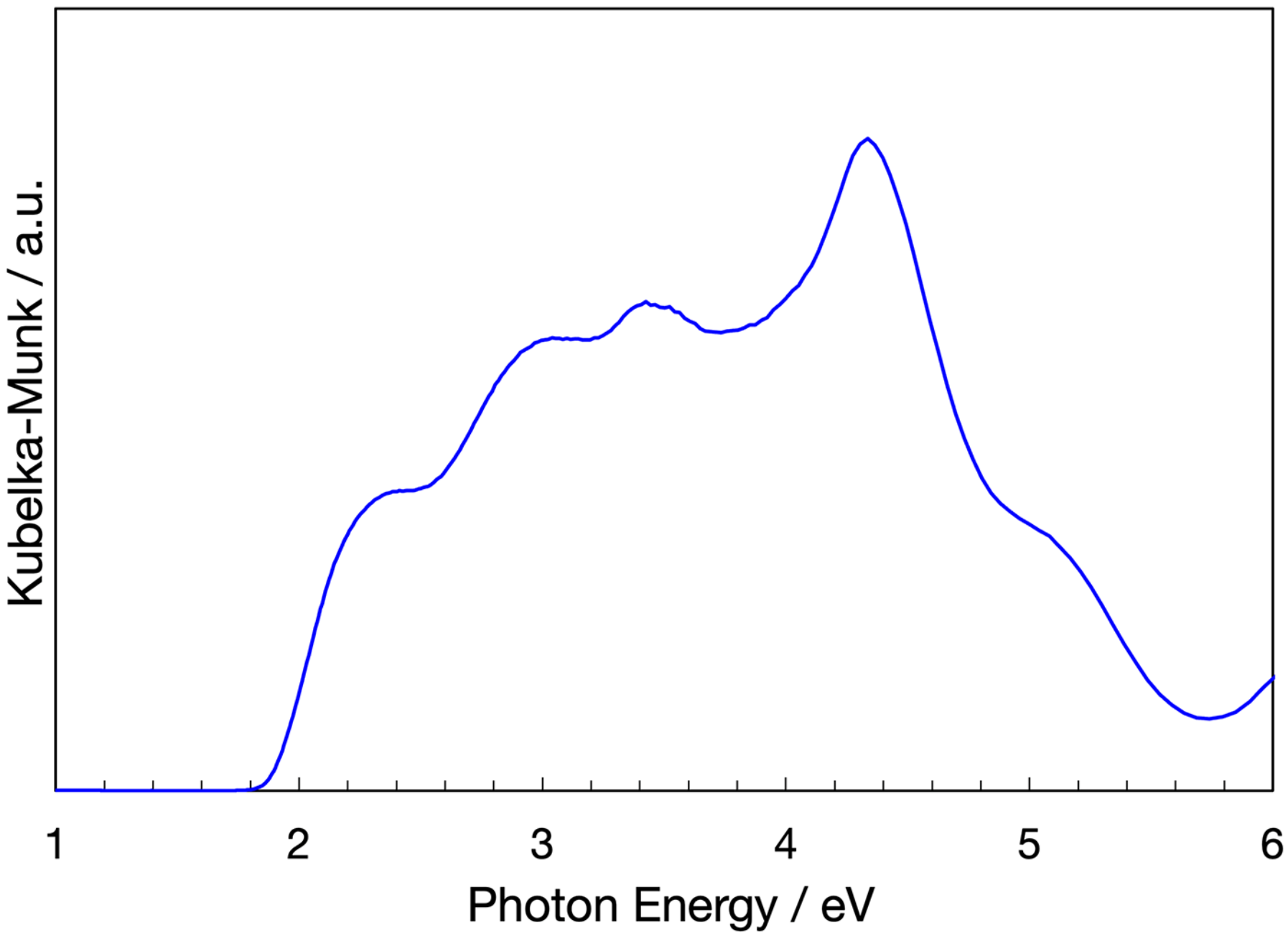

Figure S5. Diffuse reflectance spectrum of **1** at rt (same as in Figure 3c).

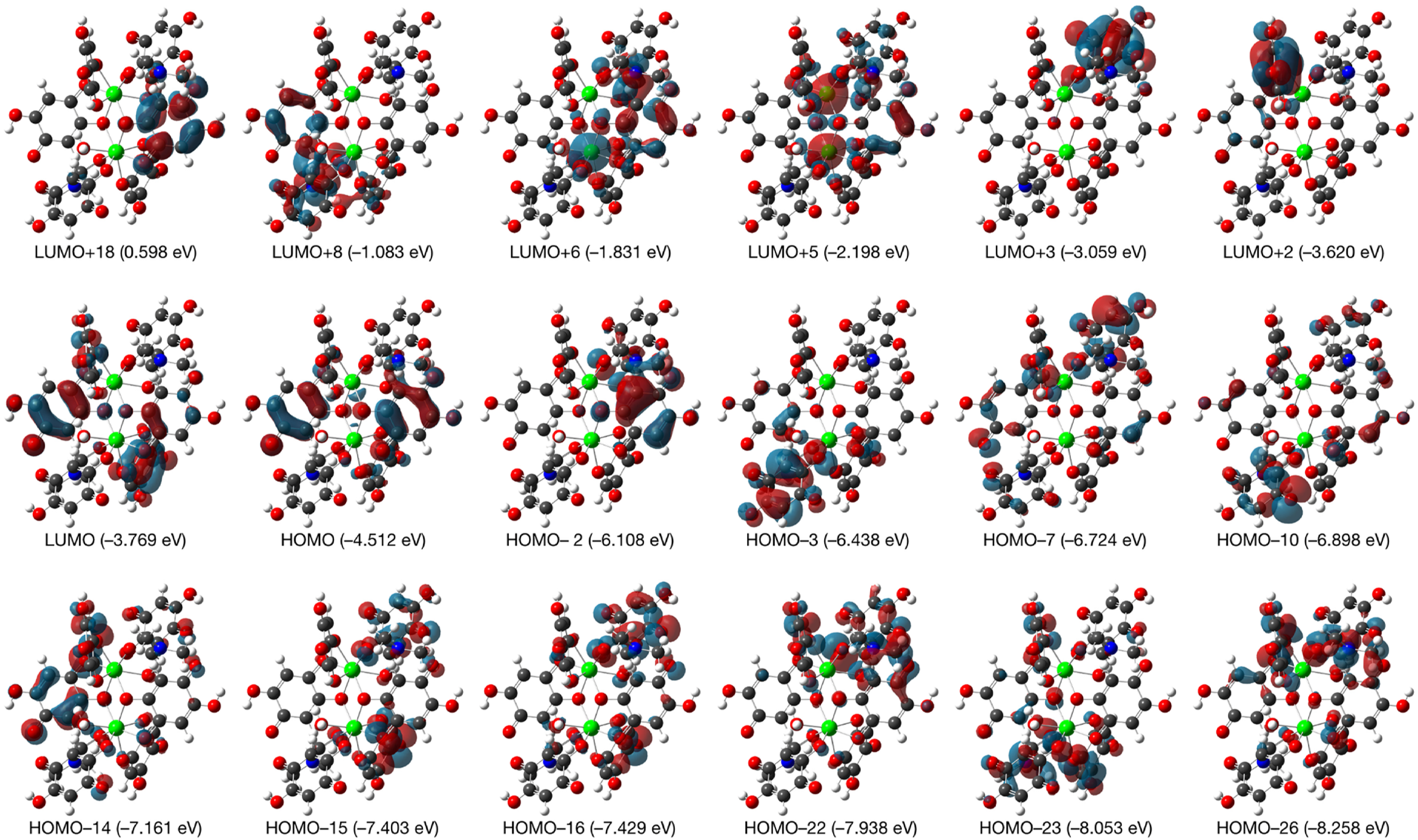

Figure S6. Selected molecular orbitals based on DFT calculation on the model structure for **1** (isovalue, 0.028 eÅ^{-3}).

Table S1. Excitation energies (*ΔE*), oscillator strengths (*f*), and assignments of excitation with relatively high probability based on the TD-DFT calculation using model structure for **1**.

***ΔE* (eV)**	***f***	**Assignment**
1.9539	0.0542	HOMO → LUMO+6 (75%) (π→sσ*, LMCT)
2.7275	0.0614	HOMO → LUMO+8 (33%) (π→pπ* (DMF))
2.7634	0.0399	HOMO–7 → LUMO+3 (25%) (π→π*)
3.0119	0.0499	HOMO–16 → LUMO (9%) (pπ (O)→π*)<br>HOMO–14 → LUMO (9%) (π→π*)<br>HOMO–14 → LUMO+2 (11%) (π→π*)
3.6444	0.0639	HOMO–23 → LUMO (20%) (pπ (DMF)→π*)<br>HOMO–22 → LUMO+3 (20%) (pπ (O)→π*)
4.3668	0.0253	HOMO–3 → LUMO+8 (48%) (π→pπ* (DMF))
4.4323	0.0561	HOMO–26 → LUMO+3 (27%) (pπ (DMF)→π*)
4.4695	0.0368	HOMO–15 → LUMO+5 (11%) (pπ (O)→sσ*, LMCT)<br>HOMO–10 → LUMO+6 (22%) (pπ (O)→sσ*, LMCT)
4.4809	0.0286	HOMO–2 → LUMO+8 (16%) (π→pπ* (DMF))<br>HOMO → LUMO+18 (22%) (π→π*)

Table S2. Cartesian coordinates for the TD-DFT calculation (based on **1**).

Center Number	Atomic Number	Atomic Type	Coordinates (Å) X	Y	Z
1	82	0	1.578440	0.529996	–0.795796
2	82	0	–1.582266	–0.537357	0.668958
3	8	0	–0.574721	0.098857	–1.187357
4	8	0	0.547096	–0.046964	1.065377
5	8	0	–2.838024	–5.988792	–2.521866
6	8	0	3.489039	5.448672	2.927447
7	8	0	–1.117918	–1.647667	2.529761
8	8	0	1.041264	1.594266	–2.672975
9	8	0	–4.151557	3.239199	–1.860118
10	8	0	4.215751	–2.990214	1.979560
11	8	0	–0.786949	–2.500379	0.048603
12	8	0	0.980208	2.160933	0.560375
13	8	0	2.845887	2.191677	–1.532322
14	8	0	–3.376759	–1.760451	1.032989
15	8	0	3.503900	0.801897	0.241520
16	8	0	–2.937585	–0.731421	–1.053556
17	6	0	–1.172877	1.274354	–1.694514
18	6	0	1.154173	–1.195153	1.645033
19	6	0	–2.942794	–5.020298	–1.721733
20	6	0	3.338069	4.711352	1.919964
21	6	0	0.230792	–2.083034	2.445566
22	6	0	–0.261779	2.099799	–2.538525
23	6	0	4.361498	4.755604	0.764576
24	6	0	–4.336340	–4.588653	–1.217743
25	6	0	–2.493228	1.672309	–1.460735
26	6	0	2.508868	–1.511301	1.505123
27	6	0	–1.792582	–4.331864	–1.292892
28	6	0	2.238100	3.846805	1.840681
29	6	0	0.700197	–3.256528	3.049987
30	6	0	–0.688586	3.288794	–3.132921
31	6	0	4.196428	3.923481	–0.357563
32	6	0	–4.455316	–3.519772	–0.318163
33	1	0	–3.136111	1.082840	–0.845813
34	1	0	3.157879	–0.872129	0.948400
35	1	0	–0.827976	–4.628377	–1.647839
36	1	0	1.522772	3.804800	2.636963
37	1	0	3.386785	–1.344335	0.010180
38	1	0	–3.286985	1.492890	0.066875
39	6	0	–2.961416	2.864738	–2.045322
40	6	0	3.002757	–2.682678	2.104150
41	6	0	–1.920394	–3.255715	–0.401856
42	6	0	2.079797	3.040774	0.707028
43	6	0	2.055544	–3.596443	2.913377
44	6	0	–2.008368	3.713027	–2.924124
45	6	0	3.089057	3.068551	–0.425857
46	6	0	–3.306635	–2.845966	0.113812
47	6	0	3.887879	–0.324693	1.029631
48	6	0	–4.034841	0.172293	–0.953463

49	1	0	3.524994	–1.304906	0.800682
50	1	0	–3.921341	1.101507	–0.442942
51	1	0	5.229288	1.789693	1.395423
52	1	0	–5.628823	–2.179119	–1.468873
53	1	0	–4.351972	–1.732772	–2.575548
54	1	0	4.500672	1.718384	2.982278
55	8	0	2.370145	–0.739562	–2.413347
56	8	0	–2.374682	0.560890	2.409245
57	1	0	0.035086	–3.887010	3.606786
58	1	0	–0.014753	3.869739	–3.733652
59	1	0	4.911843	3.943874	–1.150794
60	1	0	–5.418989	–3.220924	0.034320
61	7	0	4.688250	–0.127859	2.028702
62	7	0	–5.164406	–0.147099	–1.490340
63	6	0	3.694642	–1.190795	–2.111445
64	6	0	–3.670201	1.115228	2.142758
65	6	0	5.116467	–1.243015	2.880254
66	6	0	5.174697	1.232981	2.308532
67	6	0	–5.299609	–1.442009	–2.170224
68	6	0	–6.305403	0.776711	–1.429995
69	6	0	4.696968	–1.369643	–3.262345
70	6	0	4.081417	–1.471438	–0.791846
71	6	0	–3.998163	1.574051	0.860249
72	6	0	–4.705904	1.214663	3.280327
73	1	0	4.431431	–1.346086	3.695010
74	1	0	5.133366	–2.147427	2.309712
75	1	0	6.097116	–1.044025	3.260845
76	1	0	6.148382	1.183937	2.752641
77	1	0	–6.014596	–1.357251	–2.961170
78	1	0	–6.924976	0.635923	–2.290665
79	1	0	–6.874619	0.578253	–0.545400
80	1	0	–5.948628	1.783963	–1.406043
81	8	0	4.343880	–1.113041	–4.440541
82	6	0	5.997514	–1.811380	–2.999827
83	6	0	5.383515	–1.920513	–0.526362
84	6	0	–5.258563	2.141411	0.617110
85	6	0	–5.966320	1.778131	3.037374
86	8	0	–4.419196	0.790212	4.429613
87	6	0	6.386275	–2.095754	–1.683756
88	1	0	6.693634	–1.931610	–3.805229
89	8	0	5.734934	–2.179399	0.654157
90	8	0	–5.549728	2.564678	–0.533080
91	6	0	–6.287767	2.249770	1.757219
92	1	0	–6.681616	1.849038	3.830649
93	8	0	–2.026504	1.612751	0.459272
94	1	0	–1.805420	2.065112	1.280660
95	1	0	–1.506832	1.984565	–0.260791
96	8	0	2.024486	–1.553398	–0.279476
97	1	0	2.803499	–1.578007	0.283891
98	1	0	2.190617	–2.050506	–1.082091
99	8	0	–5.365376	–5.196075	–1.612323
100	1	0	–6.143144	–4.805535	–1.207162
101	8	0	5.343535	5.540248	0.823794
102	1	0	5.878530	5.444327	0.032479
103	8	0	2.498125	–4.644928	3.450380

104	1	0	3.435758	–4.729881	3.262690
105	8	0	–2.415005	4.782906	–3.447168
106	1	0	–3.333345	4.927776	–3.207867
107	8	0	7.556005	–2.495255	–1.447743
108	1	0	7.659856	–2.643636	–0.504982
109	8	0	–7.416108	2.762235	1.538594
110	1	0	–7.473154	3.031134	0.618790

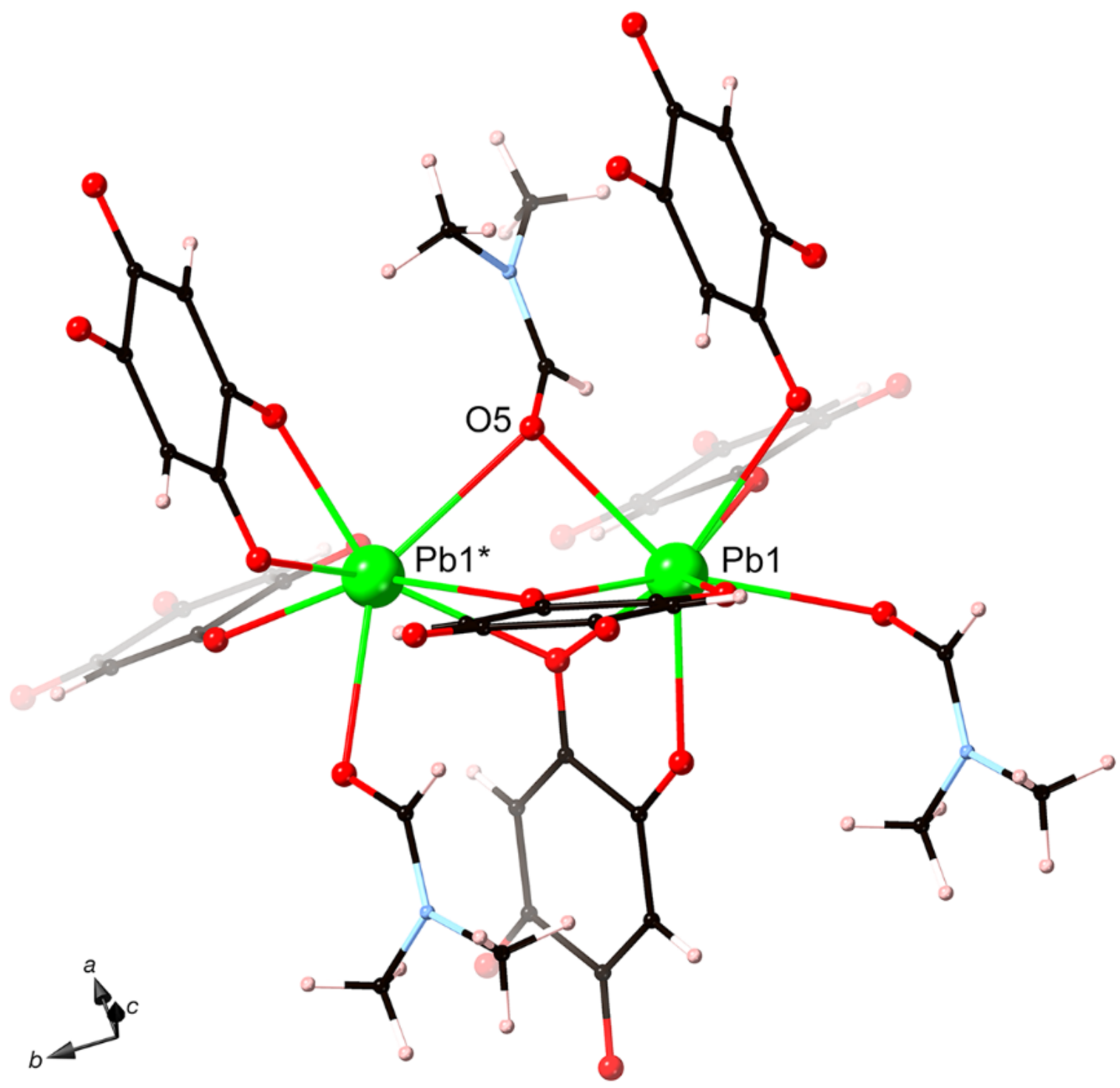

Figure S7. Bridging coordination mode of DMF molecule in **2**. Color code as in Figure 2. Pb1–O5 and Pb1*–O5 distances are 2.74, and 2.84 Å, respectively.

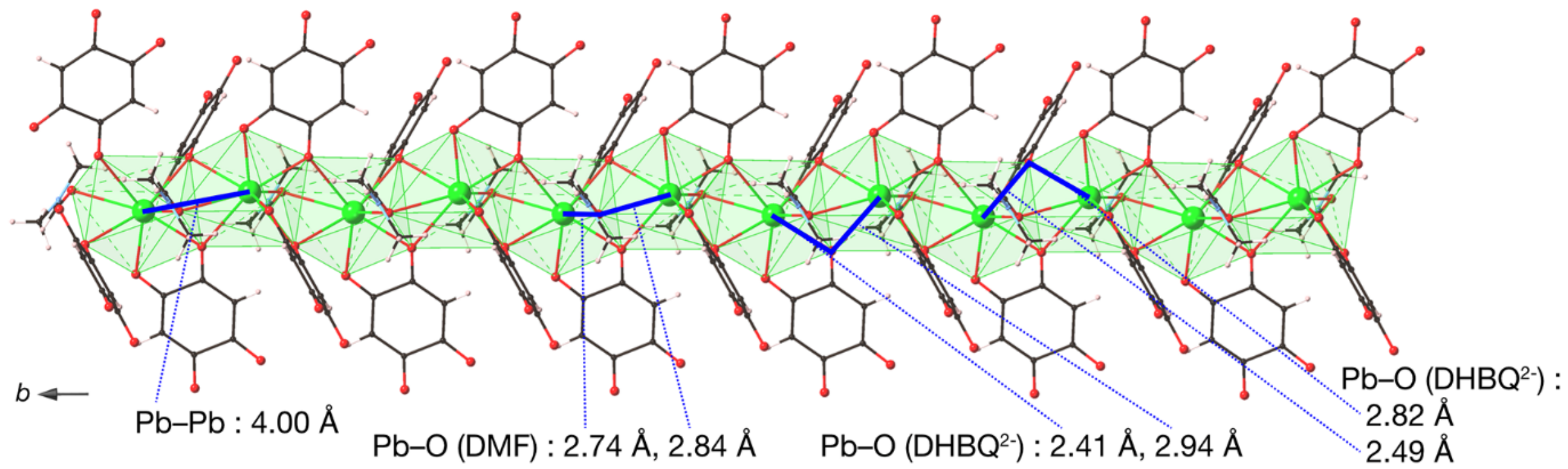

Figure S8. Pb–O structure of **2** (same as in Figure 3d). Color code as in Figure 2.

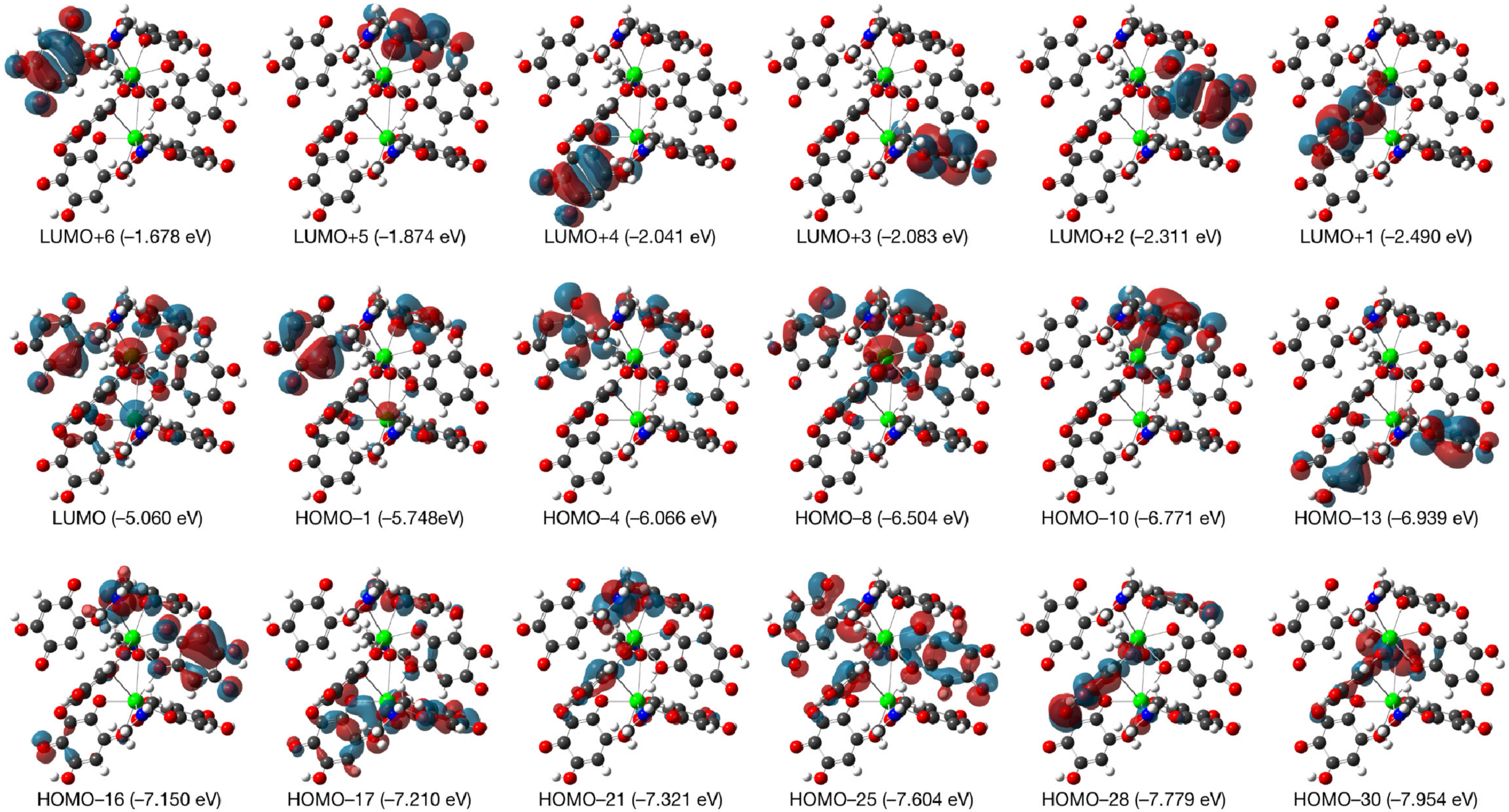

Figure S9. Selected molecular orbitals based on DFT calculation on the model structure for **2** (isovalue, 0.026 eÅ^{-3}).

Table S3. Excitation energies (*ΔE*), oscillator strengths (*f*), and assignments of excitation with relatively high probability based on the TD-DFT calculation using model structure for **2**.

***ΔE* (eV)**	***f***	**Assignment**
1.9928	0.1052	HOMO–25 → LUMO (39%) (pπ (O)→sσ*, LMCT)
2.1964	0.0619	HOMO–30 → LUMO (39%) (pπ (DMF)→sσ*, LMCT)
		HOMO–28 → LUMO (42%) (pπ (O)→sσ*, LMCT)
2.5996	0.0298	HOMO–1 → LUMO+1 (65%) (π→π*)
3.5112	0.0056	HOMO–4 → LUMO+5 (38%) (pπ (O)→π*)
4.0477	0.0629	HOMO–17 → LUMO+3 (13%) (pπ (O)→π*)
		HOMO–8 → LUMO+3 (13%) (sσ→π*, MLCT)
4.2847	0.0751	HOMO–16 → LUMO+2 (52%) (π→π*)
4.3777	0.1162	HOMO–21 → LUMO+2 (45%) (pπ (DMF)→π*)
4.3899	0.1245	HOMO–13 → LUMO+3 (14%) (π→π*)
		HOMO–8 → LUMO+6 (6%) (sσ→π*, MLCT)
4.4408	0.1908	HOMO–13 → LUMO+4 (19%) (π→π*)
4.5588	0.1369	HOMO–10 → LUMO+6 (50%) (π→π*)

Table S4. Cartesian coordinates for the TD-DFT calculation (based on **2**).

Center Number	Atomic Number	Atomic Type	Coordinates (Å) X	Y	Z
1	82	0	2.103100	–0.071958	0.354639
2	82	0	–2.086128	0.221213	0.291744
3	8	0	–0.373744	–1.584751	0.654828
4	8	0	–1.061719	–4.759468	4.723173
5	8	0	3.299322	1.850665	1.172907
6	8	0	0.663456	–5.552754	2.945106
7	8	0	–2.161257	–0.988310	2.546971
8	8	0	–0.281637	1.427518	1.514475
9	8	0	–2.807047	–1.334587	–1.969829
10	8	0	–2.846736	2.124649	1.772596
11	8	0	2.288351	–1.967042	–1.211618
12	8	0	2.222345	–0.716938	2.954860
13	8	0	–0.126715	0.888636	–1.438369
14	7	0	1.087800	–0.858359	4.897874
15	6	0	–0.489239	–2.434697	1.594162
16	6	0	–0.975466	–4.070492	3.909071
17	6	0	3.817369	2.652203	0.355768
18	6	0	0.212544	–3.599636	1.703444
19	6	0	–1.677709	–2.905429	3.800702
20	1	0	–3.225220	4.279376	3.094762
21	1	0	0.812281	–3.830486	1.030825
22	6	0	0.054500	–4.460913	2.799888
23	6	0	–1.519227	–2.044255	2.703342
24	6	0	–0.510173	2.456483	2.225165
25	6	0	–2.573930	–2.362016	–2.638065
26	6	0	0.434275	3.186377	2.911752
27	6	0	–1.385455	–3.050517	–2.539398
28	1	0	–2.553687	3.950178	–0.949757
29	1	0	–4.012352	–4.264759	–5.038777
30	6	0	–2.014586	2.846205	2.357709
31	6	0	3.331219	–2.667551	–1.149272
32	6	0	1.390947	–0.258151	3.761705
33	1	0	0.922841	0.516805	3.552574
34	6	0	1.752660	–2.094007	5.295523
35	1	0	1.408359	–2.383656	6.143112
36	1	0	1.589525	–2.771411	4.634782
37	1	0	–4.251870	–2.071533	2.808852
38	6	0	0.049532	–0.355854	5.803525
39	1	0	–0.014173	–0.931687	6.568455
40	1	0	0.277691	0.532395	6.090153
41	1	0	–0.793289	–0.337415	5.345034
42	8	0	4.105937	–1.214576	0.615758
43	8	0	3.205987	1.142646	–1.410886
44	8	0	–4.614245	–0.778623	0.489235
45	8	0	–3.529416	1.689894	–1.348865
46	6	0	–0.222643	0.434837	–2.607824
47	6	0	4.383133	3.841352	0.712676
48	6	0	3.766932	2.240855	–1.151768

49	6	0	−3.618888	−2.800046	−3.710291
50	6	0	0.108066	4.336140	3.632423
51	6	0	−1.127161	−4.201913	−3.284011
52	6	0	−2.339527	3.996058	3.077642
53	6	0	4.375125	−2.228487	−0.077370
54	6	0	3.589450	−3.817969	−1.893851
55	1	0	2.697263	−1.939069	5.369603
56	6	0	−5.558326	−0.391664	1.200893
57	6	0	−3.131579	2.518821	−2.204988
58	7	0	0.263571	1.038040	−3.676657
59	1	0	−0.688896	−0.359186	−2.742332
60	1	0	4.316376	2.867834	−2.997596
61	6	0	4.944762	4.709715	−0.234934
62	6	0	4.329102	3.110050	−2.100247
63	8	0	−4.635476	−2.104648	−3.839574
64	6	0	−3.359648	−3.951418	−4.454560
65	8	0	0.942458	5.066619	4.185571
66	6	0	−1.396437	4.725950	3.764881
67	8	0	−0.110185	−4.897333	−3.155718
68	6	0	−2.172019	−4.639939	−4.356344
69	1	0	1.319622	2.903082	2.895625
70	6	0	5.563669	−2.917018	0.021253
71	6	0	4.777108	−4.507484	−1.795583
72	1	0	6.216754	−2.604717	0.604591
73	7	0	−5.861505	−0.991975	2.337045
74	1	0	−6.026058	0.384521	0.991964
75	6	0	−2.567130	3.708931	−1.848422
76	6	0	−3.183005	2.108209	−3.712754
77	6	0	0.985020	2.300340	−3.563277
78	6	0	0.078727	0.512684	−5.033255
79	8	0	5.387483	5.656947	−0.004059
80	6	0	4.894355	4.299283	−1.742429
81	1	0	2.936750	−4.131293	−2.478092
82	1	0	4.395328	4.083682	1.610925
83	8	0	−1.591546	5.591247	4.362489
84	8	0	−1.903339	−5.504288	−4.927862
85	1	0	−2.613170	−5.726672	−5.518175
86	6	0	5.821938	−4.068453	−0.723319
87	8	0	5.098261	−5.537844	−2.476651
88	6	0	−5.196400	−2.226416	2.734821
89	6	0	−6.899554	−0.489303	3.242842
90	1	0	−0.732357	−2.737149	−1.956200
91	6	0	−2.004384	4.577258	−2.795686
92	8	0	−3.702374	0.994823	−3.983479
93	6	0	−2.620060	2.976599	−4.660967
94	1	0	1.756624	2.178086	−3.005731
95	1	0	0.410406	2.963468	−3.174492
96	1	0	1.262707	2.588367	−4.435400
97	1	0	0.467258	−0.363879	−5.092534
98	1	0	0.506626	1.095413	−5.663920
99	1	0	−0.859052	0.460003	−5.231172
100	8	0	5.412259	5.121268	−2.549348
101	8	0	6.700321	−4.669246	−0.612191
102	1	0	−5.541710	−2.516170	3.582018
103	1	0	−5.359608	−2.903874	2.074031

104	1	0	–6.963272	–1.065148	4.007763
105	1	0	–6.672345	0.398883	3.529117
106	1	0	–7.742389	–0.470876	2.784342
107	8	0	–1.484144	5.690763	–2.524633
108	6	0	–2.055703	4.165781	–4.303468
109	1	0	–2.277999	–2.674665	4.472048
110	8	0	–1.615608	4.862077	–4.987154
111	1	0	6.669405	–5.394607	–1.225069
112	1	0	5.674331	6.076580	–0.807374
113	1	0	–0.775926	5.912444	4.731234
114	1	0	–0.655796	–5.556413	4.404143
115	1	0	–1.290093	5.614530	–4.506758
116	1	0	–2.632699	2.734361	–5.558285

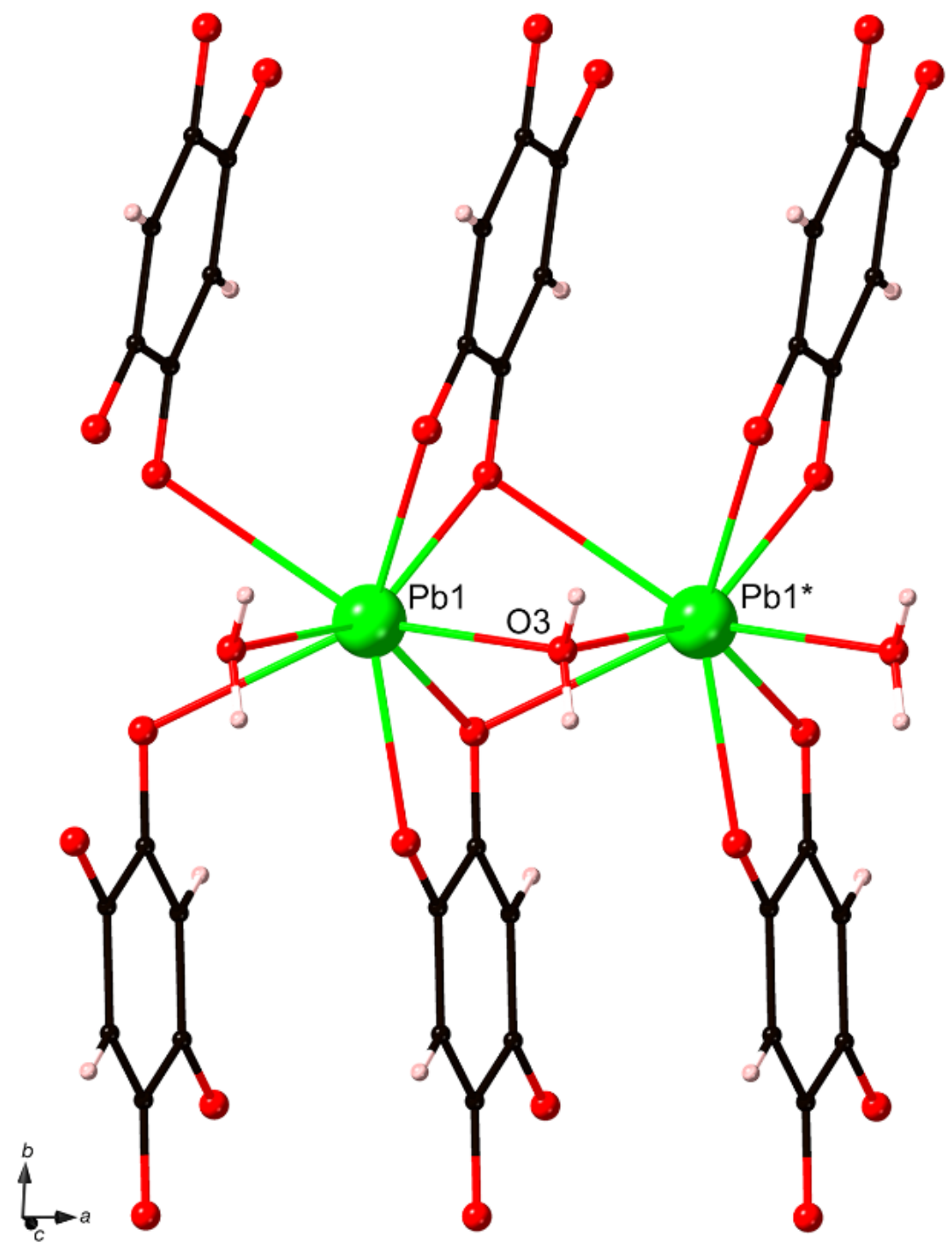

Figure S10. Bridging coordination mode of H_2O molecule in **3**. Color code as in Figure 2. Pb1–O3 and Pb1*–O3 distances are 2.53, and 2.82 Å, respectively.

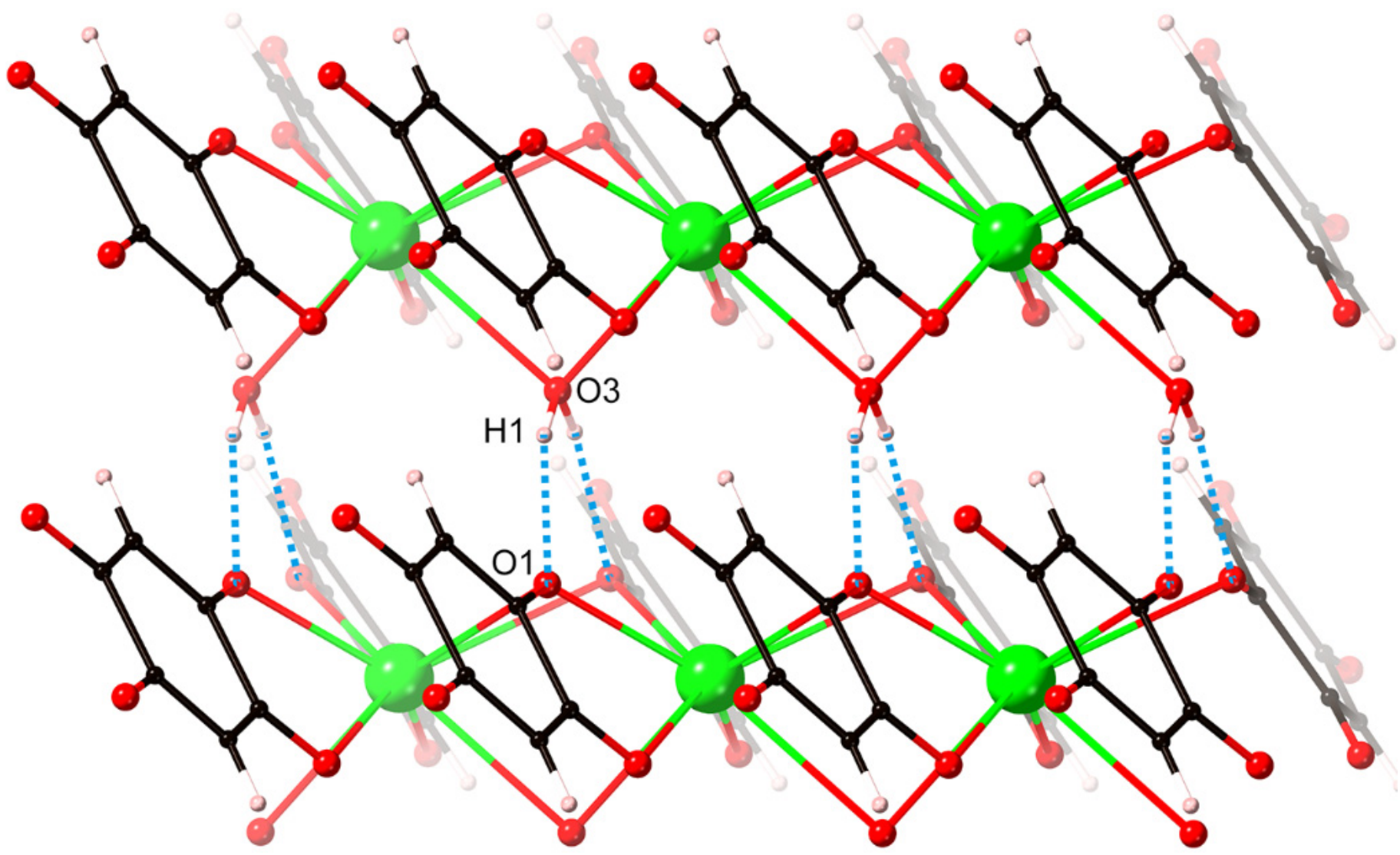

Figure S11. Hydrogen bonding in **3** between H_2O and $DHBQ^{2-}$ ligand. Color code as in Figure 2. O1–O3 distance is 2.72 Å.

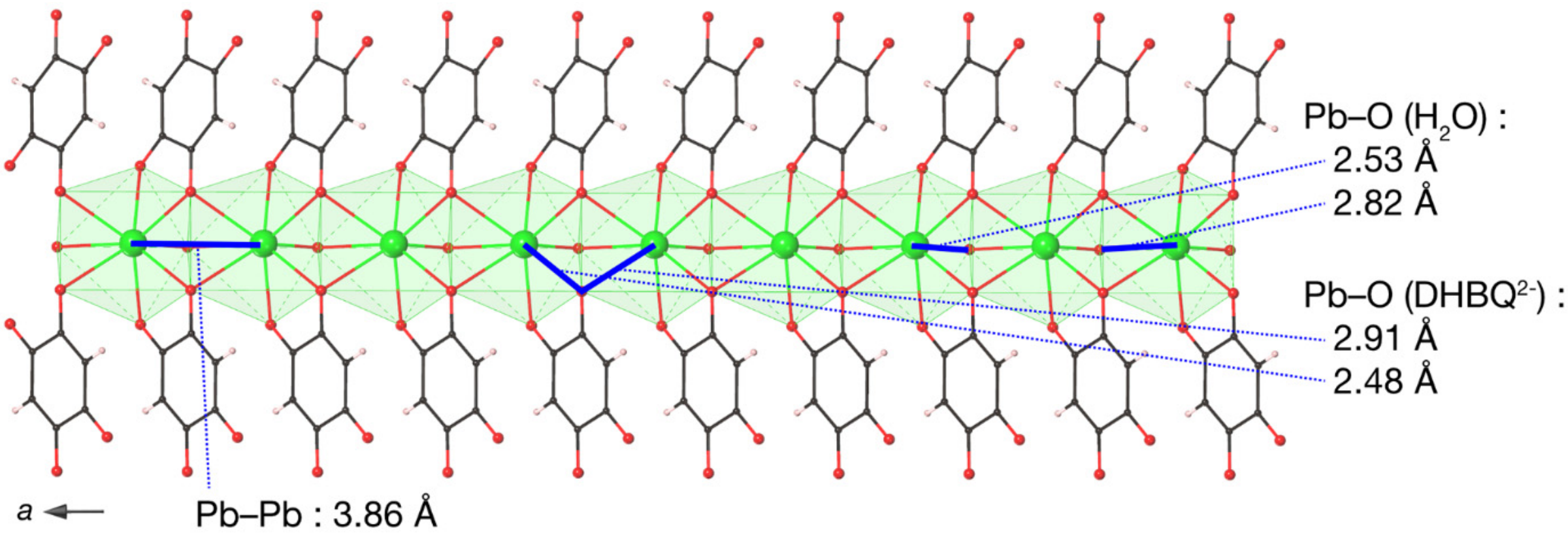

Figure S12. Pb–O structure of **3** (same as in Figure 4c). Color code as in Figure 2.

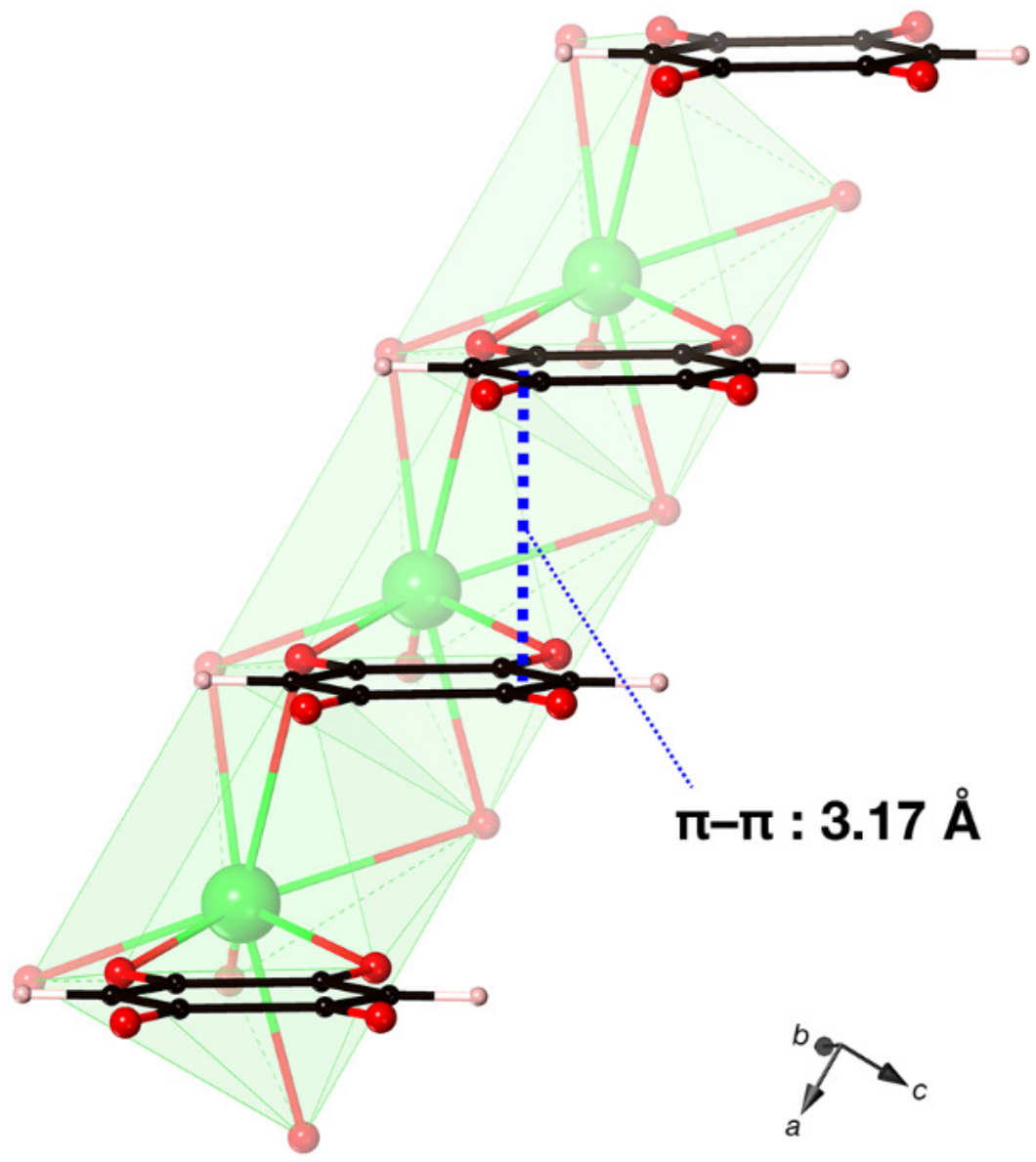

Figure S13. π–π interaction in **3**. Color code as in Figure 2.

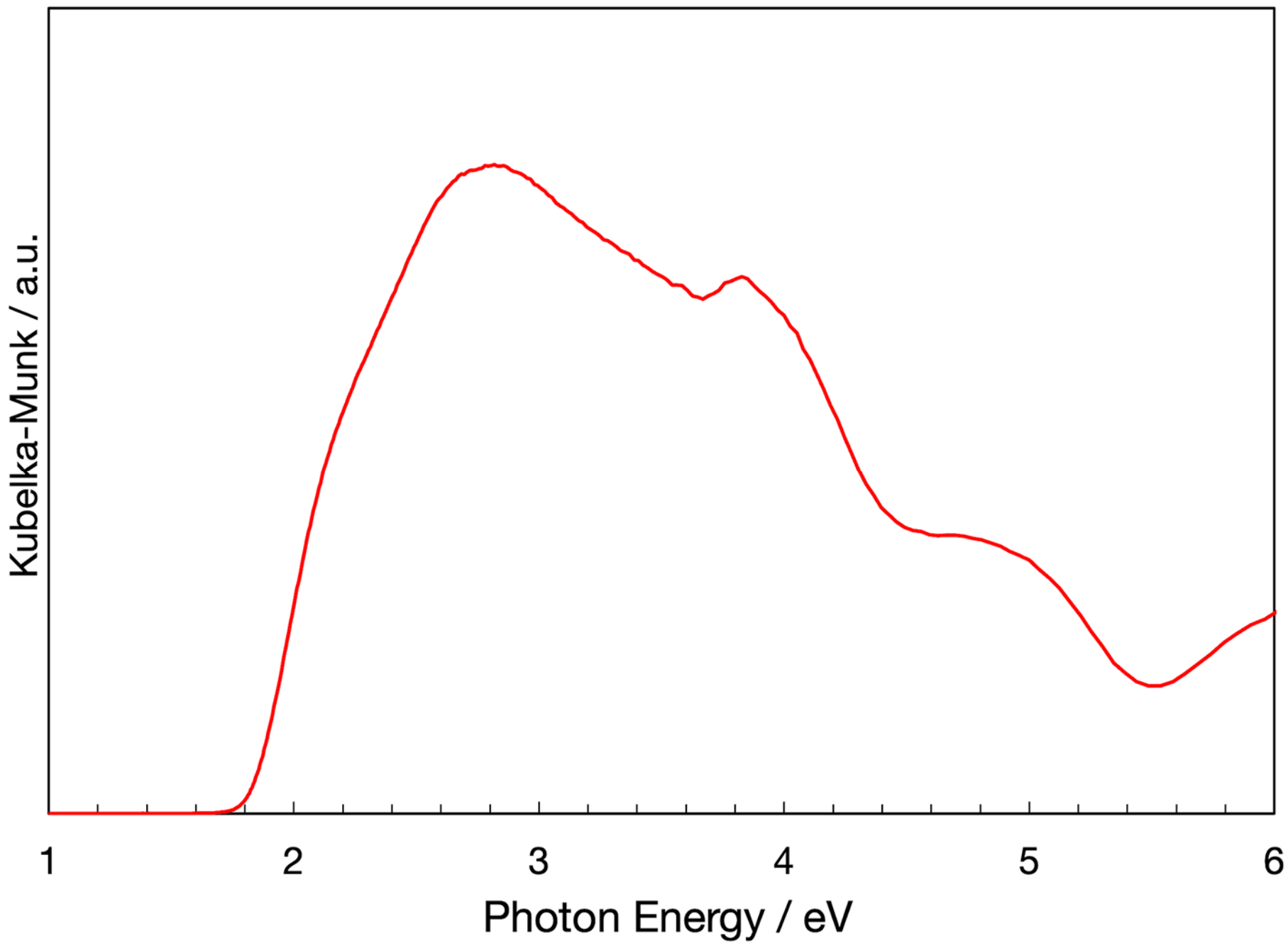

Figure S14. Diffuse reflectance spectrum of **3** at rt.

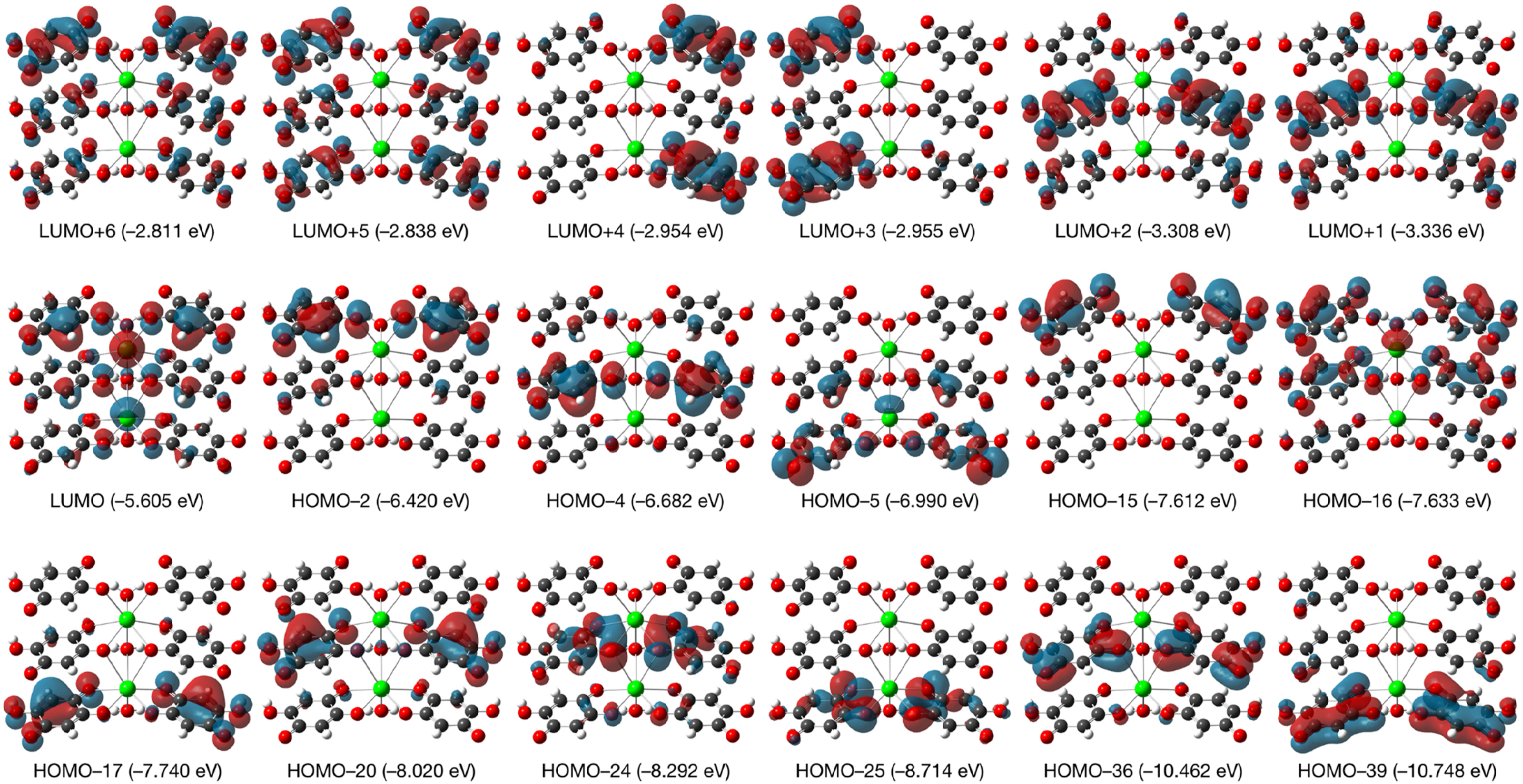

Figure S15. Selected molecular orbitals based on DFT calculation on the model structure for **3** (isovalue, 0.026 eÅ^{-3}).

Table S5. Excitation energies (*ΔE*), oscillator strengths (*f*), and assignments of excitation with relatively high probability based on the TD-DFT calculation using model structure for **3**.

***ΔE* (eV)**	***f***	**Assignment**
2.1029	0.2472	HOMO–24 → LUMO (56%) (pσ→sσ*, LMCT)
2.5844	0.0426	HOMO–25 → LUMO (33%) (pπ (O)→sσ*, LMCT)
2.6574	0.0334	HOMO–2 → LUMO+2 (14%) (π→π*)
3.0180	0.0121	HOMO–4 → LUMO+4 (15%) (π→π*)<br>HOMO–2 → LUMO+5 (17%) (π→π*)
3.1110	0.0124	HOMO–5 → LUMO+1 (31%) (pπ(O)→π*)
3.6556	0.0498	HOMO–17 → LUMO+2 (33%) (π→π*)
4.1253	0.0732	HOMO–36 → LUMO (20%) (pπ (O)→sσ*, LMCT)
4.2552	0.0673	HOMO–20 → LUMO+2 (35%) (π→π*)
4.2779	0.1361	HOMO–17 → LUMO+3 (10%) (π→π*)
4.2847	0.0822	HOMO–16 → LUMO+4 (13%) (sσ→π*, MLCT)<br>HOMO–15 → LUMO+6 (14%) (π→π*)
4.5285	0.1345	HOMO–39 → LUMO (25%) (pπ (O)→sσ*, LMCT)

Table S6. Cartesian coordinates for the TD-DFT calculation (based on **3**).

Center Number	Atomic Number	Atomic Type	Coordinates (Å) X	Y	Z
1	82	0	–0.000738	1.875827	0.039830
2	82	0	–0.000889	–2.303833	0.241035
3	8	0	1.453249	0.001606	–0.781463
4	8	0	1.464305	–3.830945	–0.567097
5	8	0	–1.452895	0.002591	–0.782371
6	8	0	–1.463859	–3.830582	–0.568206
7	8	0	2.356105	1.508610	1.167581
8	8	0	2.347998	–2.358470	1.454957
9	8	0	5.865220	–1.369901	–1.670126
10	8	0	5.865363	–5.219998	–1.393798
11	8	0	–2.355670	1.508494	1.167614
12	8	0	–2.347472	–2.357582	1.454174
13	6	0	5.479190	0.146540	0.125936
14	6	0	5.479330	–3.703571	0.402275
15	6	0	2.732986	0.009031	–0.623751
16	6	0	2.733324	–3.840008	–0.347788
17	6	0	–2.732664	0.008869	–0.623714
18	6	0	–2.732681	–3.840041	–0.347830
19	6	0	4.584160	0.875271	0.879773
20	6	0	4.584350	–2.973565	1.155821
21	6	0	3.628207	–0.719314	–1.377990
22	6	0	3.628406	–4.569224	–1.101775
23	6	0	–3.627793	–0.719647	–1.377829
24	6	0	–3.627697	–4.569636	–1.101550
25	1	0	4.879174	1.381485	1.603303
26	1	0	4.879299	–2.467717	1.879637
27	1	0	3.333206	–1.225523	–2.101527
28	1	0	3.333334	–5.075705	–1.825134
29	1	0	–3.332794	–1.225735	–2.101451
30	1	0	–3.332643	–5.075884	–1.825083
31	6	0	3.203332	0.849814	0.534379
32	6	0	3.203206	–3.000598	0.811040
33	6	0	5.009209	–0.693628	–1.033912
34	6	0	5.009345	–4.542765	–0.757627
35	6	0	–3.202963	0.849588	0.534473
36	6	0	–3.202575	–3.000805	0.811120
37	8	0	–1.481982	3.716581	–1.033652
38	8	0	1.482167	3.716825	–1.033879
39	6	0	–5.008800	–0.693982	–1.033770
40	6	0	–5.008651	–4.543123	–0.757482
41	6	0	–4.583809	0.874953	0.879897
42	6	0	–4.583703	–2.973948	1.155996
43	6	0	–2.732933	3.859640	–0.900798
44	6	0	2.733046	3.859818	–0.900862
45	6	0	–5.478807	0.146145	0.126097
46	8	0	–5.864780	–1.370323	–1.669954
47	6	0	–5.478663	–3.703962	0.402433
48	8	0	–5.864638	–5.220420	–1.393626

49	1	0	–4.878835	1.381129	1.603449
50	1	0	–4.878679	–2.468047	1.879764
51	6	0	–3.202957	4.698263	0.258622
52	6	0	–3.627920	3.129099	–1.653760
53	6	0	3.203011	4.698498	0.258528
54	6	0	3.628035	3.129372	–1.653880
55	6	0	–4.583969	4.725130	0.603495
56	8	0	–2.346994	5.375827	0.895612
57	6	0	–5.008942	3.156107	–1.310092
58	1	0	–3.332963	2.623521	–2.377827
59	8	0	2.347024	5.375995	0.895545
60	6	0	4.584037	4.725461	0.603360
61	1	0	3.333094	2.623754	–2.377920
62	6	0	5.009067	3.156468	–1.310239
63	6	0	–5.478951	3.995256	–0.150167
64	1	0	–4.878974	5.230208	1.327209
65	8	0	–5.864923	2.478776	–1.946210
66	1	0	4.879027	5.230559	1.327066
67	6	0	5.479050	3.995650	–0.150328
68	8	0	5.865078	2.479198	–1.946382
69	8	0	–6.749660	–3.697066	0.638545
70	1	0	–6.932227	–3.133372	1.393872
71	8	0	6.750335	–3.696581	0.638347
72	1	0	6.932883	–3.132874	1.393669
73	8	0	6.750192	0.153516	0.362020
74	1	0	6.932802	0.717658	1.117002
75	8	0	6.750050	4.002616	0.085763
76	1	0	6.932598	4.566322	0.841085
77	8	0	–6.749945	4.002130	0.085961
78	1	0	–6.932512	4.565824	0.841288
79	8	0	–6.749802	0.153031	0.362217
80	1	0	–6.932430	0.717159	1.117205
81	1	0	0.797617	–3.570035	2.952014
82	1	0	–0.769303	–3.532824	2.981904
83	8	0	0.002372	–3.593375	2.406712
84	1	0	–0.786457	3.981156	2.436423
85	1	0	0.786997	3.981664	2.435813
86	1	0	0.784219	0.248273	2.702616
87	1	0	–0.783759	0.248141	2.702659
88	8	0	0.000213	0.126562	2.144543
89	8	0	0.000425	3.405442	2.386350

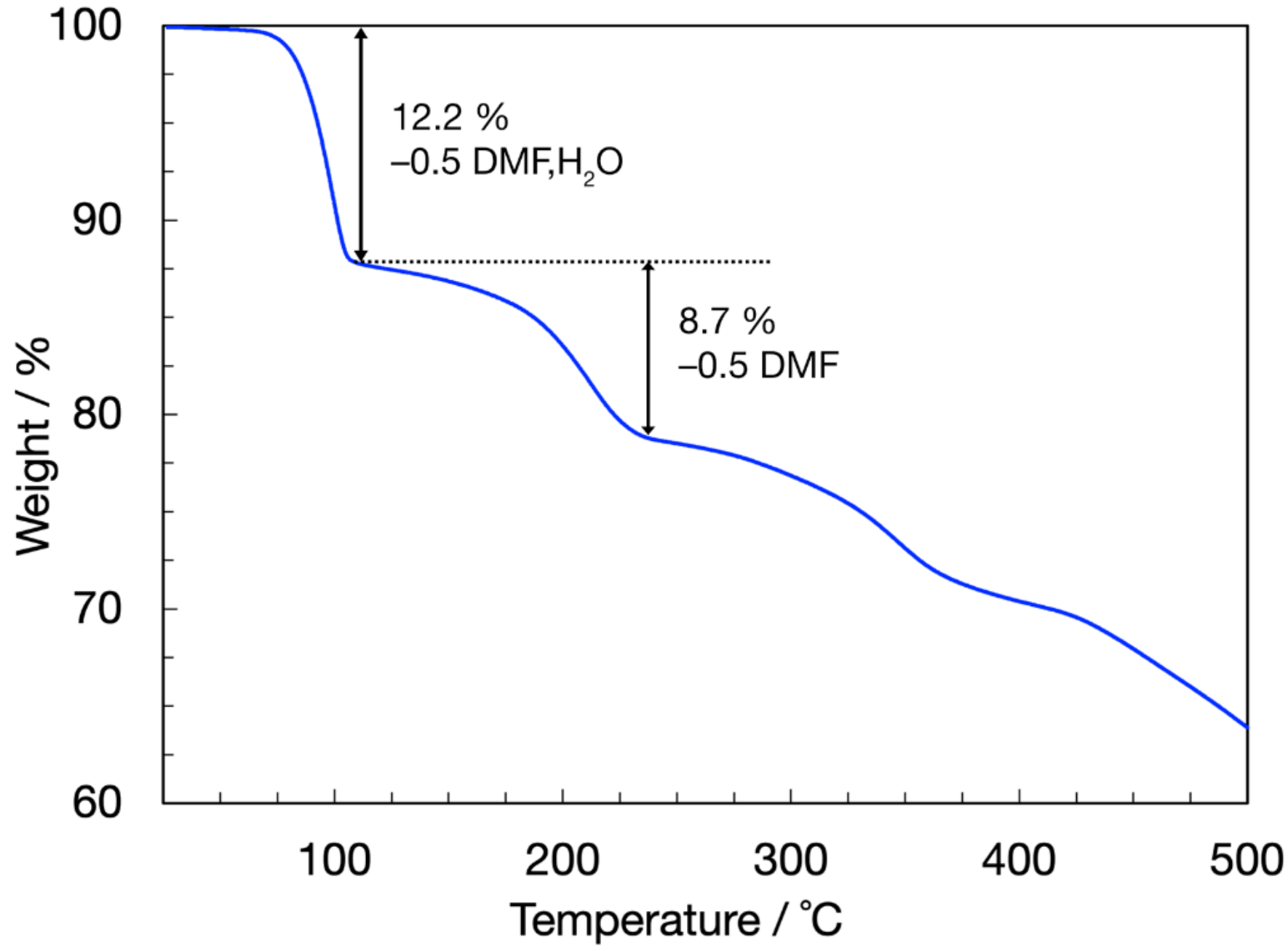

Figure S16. TGA curve of **1** under N_2 flow. Assignments of weight loss based on the elemental analysis are also shown.

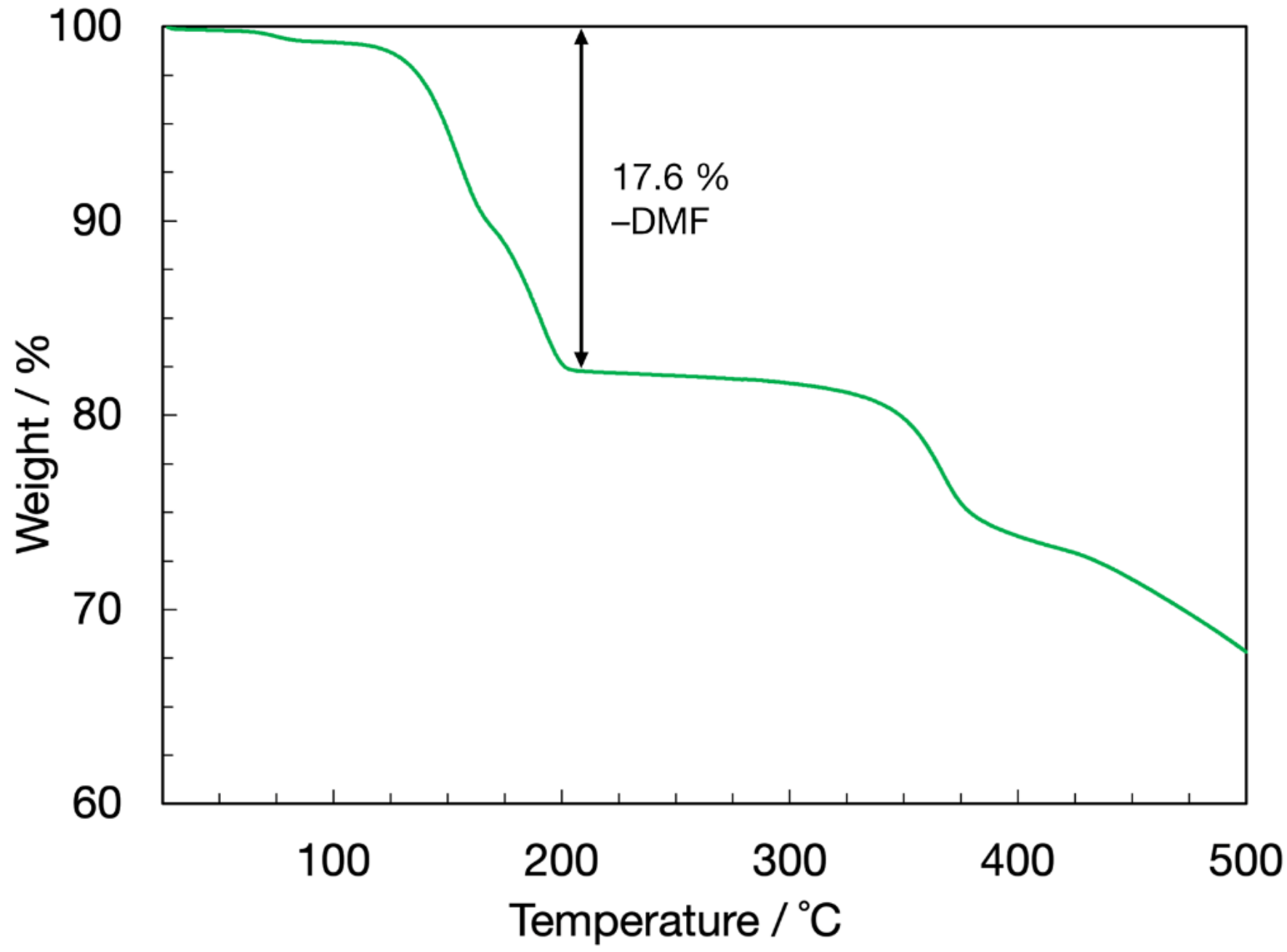

Figure S17. Same as Figure S16 but for **2**.

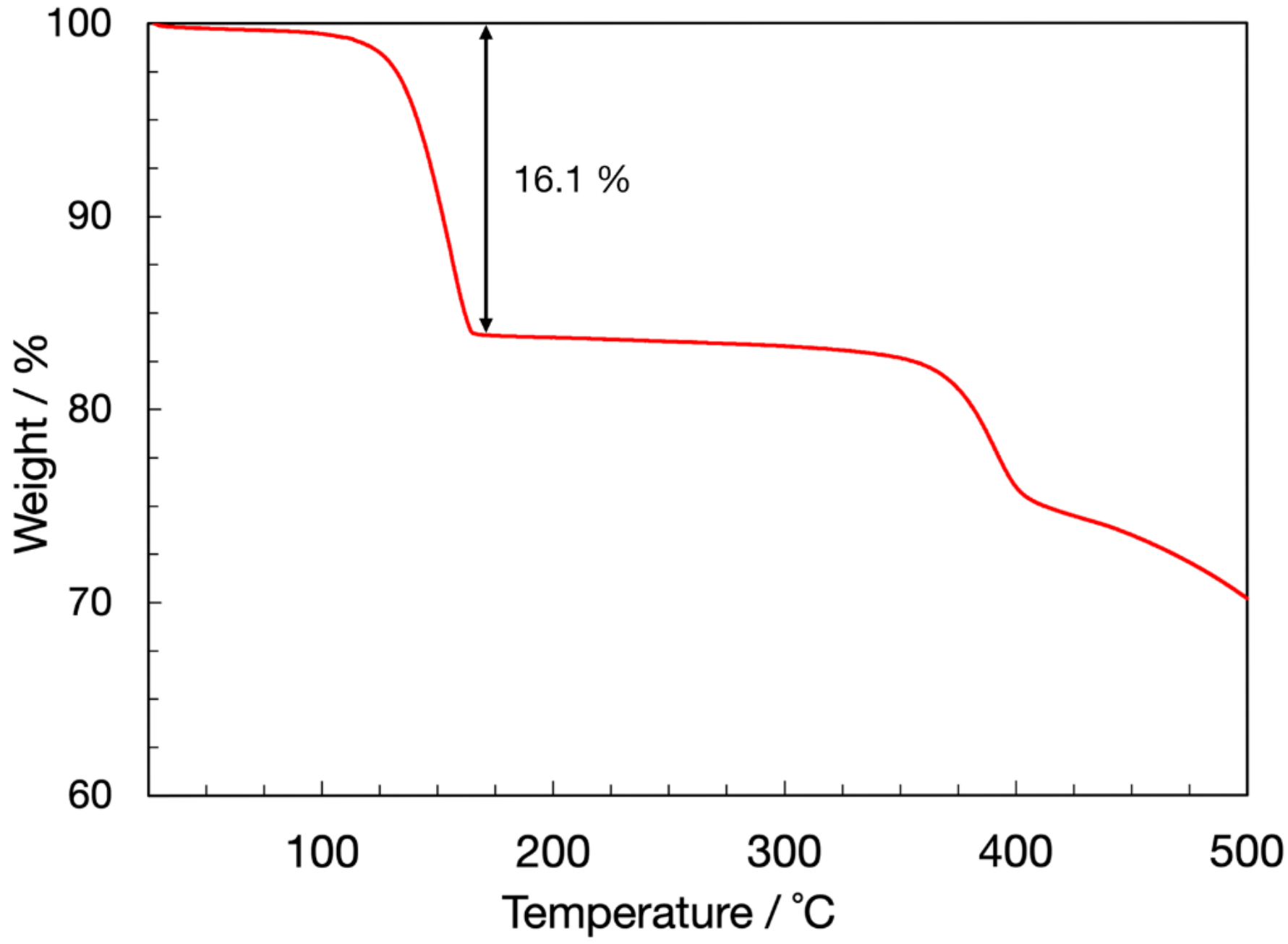

Figure S18. Same as Figure S16 but for **3**. For compound **3**, the first weight loss at around 123 °C (16.1 %) can not be assigned to the simple loss of the coordinative H_2O molecules, suggesting partial decomposition of the crystal structure.

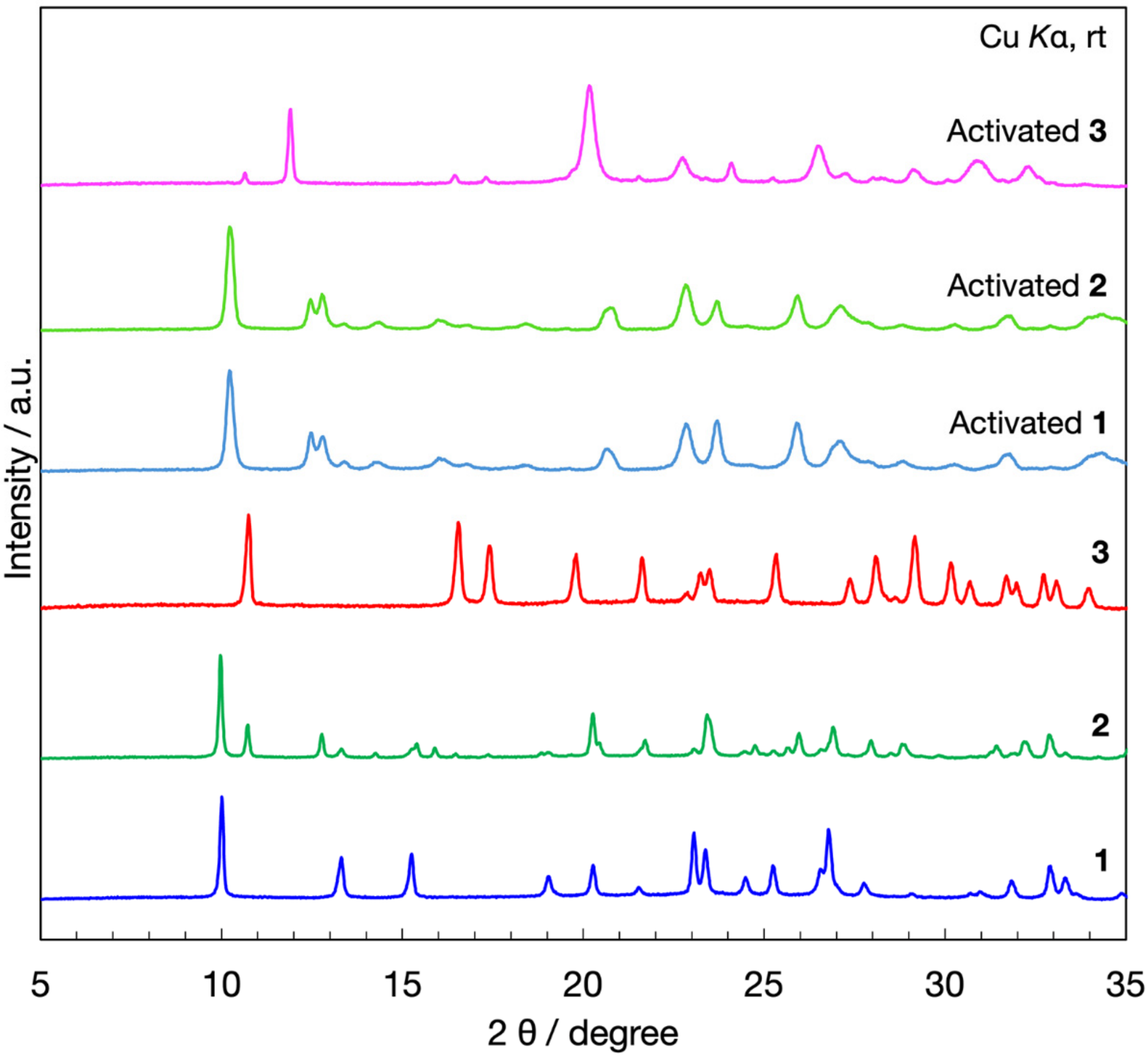

Figure S19. PXRD patterns of **1**–**3** and their activated phases. Activation conditions were determined according to the TGA data (Figures S16–S18). Activation conditions for each compound are as follows: Heating at 220 °C for 2.5 h (**1**), Heating at 200 °C for 2.5 h (**2**), and Heating at 180 °C for 2.5 h (**3**). As clearly seen, activation of **1** and **2** resulted in the formation of the same desolvated phase possessing an identical crystal structure, whereas activation of **3** provided a mixture of multiple crystalline phases with different crystallite diameters.

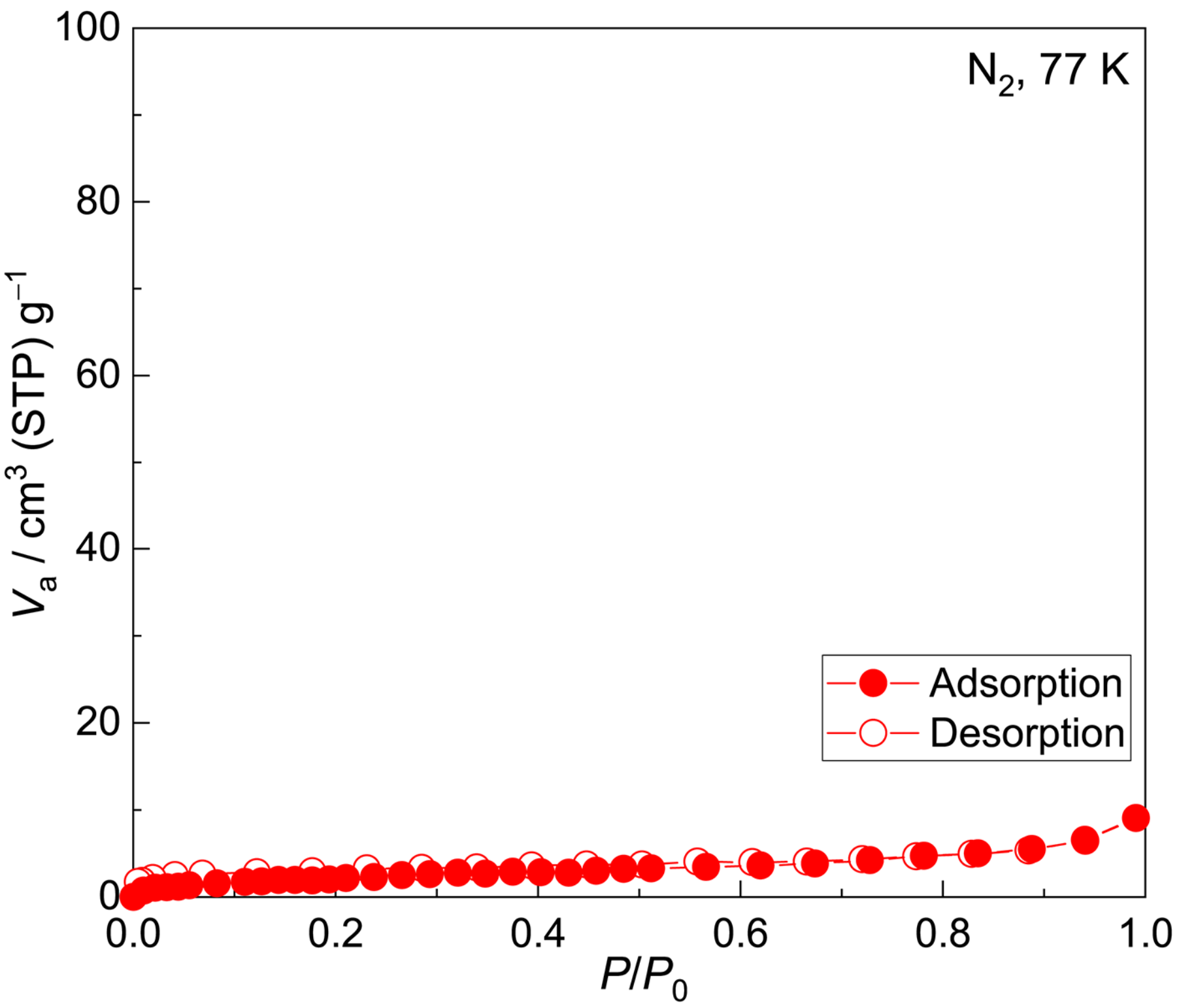

Figure S20. N_2 adsorption isotherm of activated form of **1** at 77 K.

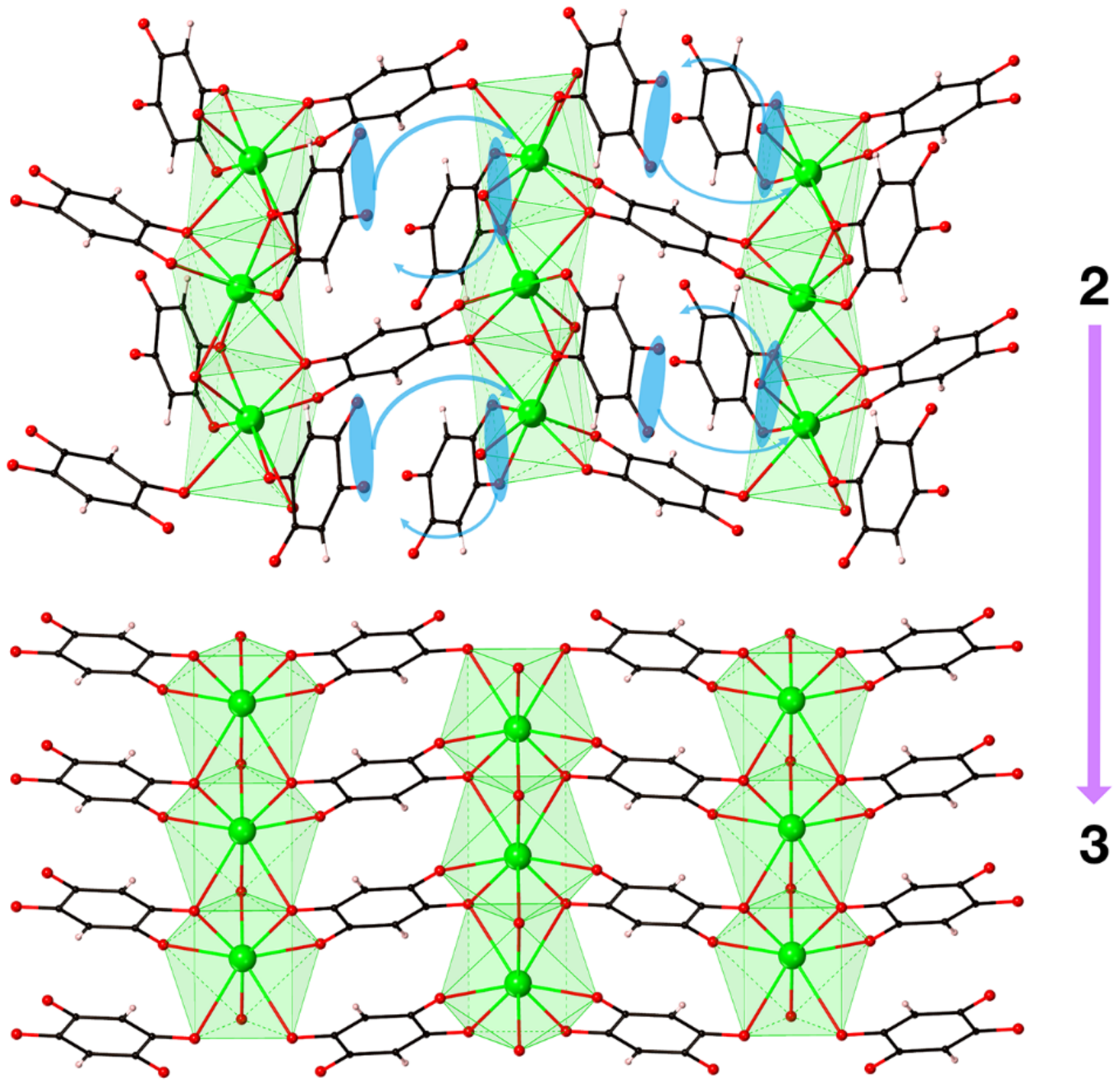

Figure S21. Possible structural rearrangement during transformation from **2** to **3**. Color code as in Figure 2. Possible rearrangement directions are highlighted in light blue (**2**). As a result of the rearrangement of $DHBQ^{2-}$ ligand, effective π–stacking is formed (**3**).
